# CRISPR/Cas9: an advanced platform for root and tuber crops improvement

**DOI:** 10.3389/fgeed.2023.1242510

**Published:** 2024-01-19

**Authors:** K. Divya, Makeshkumar Thangaraj, N. Krishna Radhika

**Affiliations:** ICAR-Central Tuber Crops Research Institute, Thiruvananthapuram, India

**Keywords:** root and tuber crops, CRISPR/Cas9, genome editing, double-stranded breaks, targeted editing, editing efficiency, genetic transformation

## Abstract

Root and tuber crops (RTCs), which include cassava, potato, sweet potato, and yams, principally function as staple crops for a considerable fraction of the world population, in addition to their diverse applications in nutrition, industry, and bioenergy sectors. Even then, RTCs are an underutilized group considering their potential as industrial raw material. Complexities in conventional RTC improvement programs curb the extensive exploitation of the potentials of this group of crop species for food, energy production, value addition, and sustainable development. Now, with the advent of whole-genome sequencing, sufficient sequence data are available for cassava, sweet potato, and potato. These genomic resources provide enormous scope for the improvement of tuber crops, to make them better suited for agronomic and industrial applications. There has been remarkable progress in RTC improvement through the deployment of new strategies like gene editing over the last decade. This review brings out the major areas where CRISPR/Cas technology has improved tuber crops. Strategies for genetic transformation of RTCs with CRISPR/Cas9 constructs and regeneration of edited lines and the bottlenecks encountered in their establishment are also discussed. Certain attributes of tuber crops requiring focus in future research along with putative editing targets are also indicated. Altogether, this review provides a comprehensive account of developments achieved, future lines of research, bottlenecks, and major experimental concerns regarding the establishment of CRISPR/Cas9-based gene editing in RTCs.

## 1 Introduction

Root and tuber crops (RTCs), the plants that store carbohydrates in subterranean roots/corms/rhizomes/tubers, are the second largest cultivated species, after cereals, in tropical countries and have a significant role in global food security. RTCs provide a dietary supplement for 2.2 billion people in developing countries (Tussipkan and Manabayeva, 2021). Cassava [(*Manihot esculenta* Crantz, Family: Euphorbiaceae], sweet potato [(*Ipomoea batatas* (L.), Family: Convolvulaceae)], potato (*Solanum tuberosum*), yams (*Dioscorea* spp., Family: Dioscoreaceae), and aroids [elephant foot yam (*Amorphophallus paeoniifolius*), taro (*Colocasia esculenta*), giant taro [*Alocasia macrorrhiza* (L.) Schott], tannia or yautia (*Xanthosoma sagittifolium*), and swamp taro [(*Cyrtosperma chamissonis* (Schott) Merr.), Family: Araceae] are the major RTCs. Chinese potato [(*Solenostemon rotundifolius* (Poir.) J.K. Morton), Family: Labiatae], arrowroot [(*Maranta arundinacea* L.), Family: Marantaceae], yam bean [(*Pachyrhizus erosus* (L.) Urban, Family: Leguminosae], and canna [(*Canna edulis* (Ker-Gawler), Family: Cannaceae] are minor RTCs. Potatoes contribute to 44% of the global RTC production, followed by cassava (32.91%), sweet potatoes (12.72%), yams (8.23%), and aroids (2.4%) (Jaganathan et al., 2020). The largest global producer of RTCs is Africa, followed by Asia, Europe, and America (FAOSTAT, 2019). In 2017, the global production of RTCs was 494.6 million tons (Tussipkan and Manabayeva, 2021). Additionally, RTCs considerably contribute to the financial stability of local growers and the associated population through direct sales and value addition. Characteristic features of RTCs like production of a large amount of edible energy per hectare per day, low cost of cultivation, minimum agricultural input, and wide adaptability to diverse environmental and soil conditions and agricultural practices and expanding opportunities for value addition and industrial applications make them promising crops for sustainable agriculture.

Prevailing improvement strategies for RTCs are insufficient in many aspects. Conventional methods like hybridization, mutation breeding, marker-assisted breeding, and genetic engineering approaches for developing new RTC varieties confront a multitude of challenges that are extremely difficult to deal with. Thus, there is an urgent necessity for the evaluation and implementation of innovative technologies for RTC improvement. The successful application of genome editing tools from meganucleases to recently evolved clustered regularly interspaced short palindromic repeats/CRISPR-associated protein (CRISPR/Cas) for creating desirable traits in various crop species is well known. The gene editing technique makes precise changes in the genome of living organisms by inducing heritable targeted mutations at specific genomic sites.

CRISPR/Cas9 is a third-generation gene editing technique that gained quick popularity owing to its easily customizable and flexible design, simple operation strategy, high precision, and efficient multiplexing ability. In this RNA-guided engineered nuclease (RGEN) system, a 20-nt RNA sequence directs Cas9 endonuclease to the target genomic site. Cas makes double-stranded breaks (DSBs) in the target DNA. DSBs are repaired by cellular DNA damage repair mechanisms, error-prone non-homologous end-joining (NHEJ), and high-fidelity homology-dependent repair (HDR) (Gorbunova and Levy, 1999). NHEJ creates random insertions, deletions, substitutions, inversions, and translocations. The HDR mechanism is mostly restricted to dividing cells and requires a repair template such as single-stranded or double-stranded DNA with homology arms (Puchta, 2005; Soyars et al., 2018). This pathway enables gene replacement or knock-ins and protein-domain swapping. The cell cycle phase and nature of the DSB end determine the choice of the repair pathway.

Although reviews on CRISPR-based editing are available, a focus on different aspects like improvement, value addition, protection, and utilization of major RTCs where CRISPR-based gene editing can have an impact is described for the first time in this review. The review gives a comprehensive overview of the gene editing work done so far in RTCs along with interesting areas to work up for the improvement and utilization of major RTCs and many underutilized RTCs.

## 2 The CRISPR/Cas mechanism

Naturally, CRISPR/Cas is a part of the bacterial and archaeal adaptive immune system. The bacterial CRISPR/Cas system comprises 25–40 bp variable spacer sequence acquired from invading nucleic acids, CRISPR-associated (Cas) genes, leader sequence, distinctive array of conserved repetitive elements of 14–21 bp interspaced between spacer sequence (Ishino et al., 1987). The spacer sequence, that is, the remnants of past invasion act as memory and recognition elements in the host which trigger an immune response upon a second encounter by guiding the Cas endonuclease to the invader’s genome, where Cas makes sequence-specific cleavage and thereby inactivates the same in a three-step mechanism—adaptation, expression, and interference (Barrangou, et al., 2007; Bhaya et al., 2011; Charpentier and Doudna, 2013). To manipulate the bacterial CRISPR system for editing purposes, the natural dual RNA structure formed of target-specific CRISPR RNA (crRNA) and trans-activating CRISPR RNA (tracrRNA) has been simplified into a single guide RNA (sgRNA) of ∼100 bp. The sgRNA comprises conserved sequences and a structure of duplex that is inevitable for targeting activities and a target-specific 20-nt guide RNA (gRNA) in the 5′ of this gRNA scaffold (Jinek, et al., 2012; Xing et al., 2014). Any genomic loci can be easily targeted by merely altering the 20-nt gRNA at the 5′ of sgRNA.

Target recognition requires the presence of a conserved common end sequence, a specific di-nucleotide downstream of the target sequence, known as protospacer adjacent motif (PAM) or CRISPR motif, which is different for each Cas enzyme (Jinek, et al., 2012; Doudna, J. A., and Charpentier, E. 2014). PAM for the most used *Streptococcus pyogenes* Cas9 (SpCas9) is 5′-NGG-3′. The specificity of target site identification and Cas9 cleavage depends on a contiguous stretch of 12–13 bp upstream of PAM in the target sequence which is known as the seed sequence/seed region. Twelve base pairs from the 3′-end of gRNA should be exactly complementary to the seed region for avoiding off-target cleavage activities (Semenova, et al., 2011; Jinek, et al., 2012; Mali et al., 2013; Xie and Yang, 2013; Fu et al., 2014; Sander and Joung, 2014). A strict requirement for PAM sequence limits the targets for editing and this evoked the evaluation of alternative Cas endonucleases with flexible PAM specifications that could expand the range of the targetable sequences. Cpf1 or Cas12a from *Prevotella* and *Francisella* species (Makarova et al., 2015; Zetsche et al., 2015), Cas9 from *Francisella novicida* (FnCas9) (Zhang et al., 2018), and Class 2-type VI-A CRISPR/Cas effector from *Leptotrichia shahii* (LshCas13a) or *Leptotrichia wadei* (LwaCas13a) (Abudayyeh et al., 2017) can be potential alternatives in this regard.

According to the recent classification, there are two classes, six types, and 19 subtypes of CRISPR systems each of which with a signature Cas protein. The Class 2, type II system is considered to be the most suitable one for genome editing applications owing to the requirement for a single Cas enzyme, namely, Cas9. Cas9 solely performs the complete operation of editing events, unlike the other systems with several distinct multi-subunit enzyme complexes (Makarova et al., 2011; Zetsche et al., 2015). The type II CRISPR/Cas9 system in *S. pyogenes* is the most studied and widely used CRISPR/Cas system (Jinek, et al., 2012; Bortesi and Fischer, 2015). As there is no need for engineering new DNA-protein interfaces for each different target sequence, RNA-directed DNA targeting by CRISPR/Cas9 is less complicated and enables high-throughput multiplex editing. Multiplexing is possible with Pol III promoter-sgRNA units or using a tRNA-sgRNA single transcription unit (STU) system. Apart from gene knockouts and knock-ins, CRISPR/Cas9 expands its applications to various dimensions like gene expression regulation, epigenetic reprogramming, chromatin visualization, and remodeling. This is facilitated primarily by Cas9 with mutated HNH and RuvC domains, i.e., nuclease-deficient Cas9 or dead Cas9 (dCas9), which lacks cleavage activity but retains target recognition and binding ability (Qi et al., 2013; Gilbert et al., 2014). Chimeric protein complexes generated by the fusion of the C-terminus of dCas9 with different effector domains, i.e., transcriptional activators or repressors or reporter proteins, can be employed as tools for transcriptional regulation and DNA visualization (Cheng et al., 2013; Maeder et al., 2013).

## 3 The need for genome editing for the improvement of RTCs

Enrichment of desirable traits and elimination of unwanted characteristics in RTC species is required for the full-fledged utilization of their nutritional values and industrial potential. Low productivity, loss due to diseases and pests, poor quality of planting material, limited shelf life which is mostly caused due to high moisture content, undesirable or unpalatable/anti-nutritional/toxic components, bulky nature, space consumption, expensive transportation, perishability of harvested products, and non-uniform tuber shape that creates difficulties in mechanical peeling are the major constraints associated with cultivation and consumption of RTCs (Lebot, 2010; Sanginga and Mbabu, 2015).

Erratic flowering, low fertility and seed set rate, heterozygosity, variable ploidy levels, self-incompatibility, long life cycle, dioeciousness as in the case of yam species, the absence of commercial seed industry, limited availability of improved varieties, and a lack of variation in the available germplasm all render breeding programs for RTC improvement very difficult, less efficient, and time-consuming. Farming practices like clonal propagation of infected planting material cause severe yield loss (Lebot, 2010).

Novel breeding techniques can address these issues and provide solutions to enable large-scale cultivation, efficient post-harvest management, easy processing, long-distance transport, long-term storage, and value addition of RTCs. The recombinant DNA technology which enables the introduction or replacement and knockout of any gene of interest can be utilized for the improvement of RTCs. However, the biosafety regulations associated with the cultivation and commercialization of genetically modified plants limit the field-level application of such methods.

Under such circumstances, new breeding techniques like genome editing, based on programmable nucleases and host DNA repair mechanisms can be a potential strategy for the improvement of RTCs. The ability of this technique to unravel the trait-determining genes and interwoven genetic regulatory networks by sequence-specific mutagenesis and for creating novel as well as improved phenotypes by incorporating or modifying specific genes in an efficient, precise, and simple manner opens new avenues for RTC improvement and thereby helps safeguard global food security.

## 4 CRISPR/Cas9 for RTC improvement

A multidimensional genome editing platform created by CRISPR/Cas9 provides opportunities for rewriting the genome of RTC species to attain a wide range of goals in nutritional and economic contexts. The predominant objectives that are envisaged to be fulfilled by the CRISPR/Cas9 genome editing toolbox include biofortification, alleviation of toxicity in certain tubers, reinforcement of stress tolerance mechanisms, breaking barriers in breeding, reprogramming of certain developmental processes/patterns, customization of metabolism or structural and biochemical composition, and remodeling of post-harvest tuber metabolism. The genomic target to be mutated varies according to the required trait modification. Functional annotation of putative genes by precisely targeted mutagenesis would reveal the trait-determining genes that can be modified by employing CRISPR/Cas9. Intrinsic genes conferring favorable traits can be activated, and those encoding undesirable traits can be knocked out or repressed. The incorporation of desirable traits can also be achieved by inducing HDR-mediated DSB repair. Varieties compatible with taste and market preferences may not be fit for cultivation when important agronomic traits are considered. This emphasizes the importance of having varieties possessing good agronomical traits and desirable characteristics suitable for multiple purposes. So, genome editing–based crop improvement programs that are focused on farmer-preferred landraces will be more helpful for their field-level application.

**FIGURE 1:**
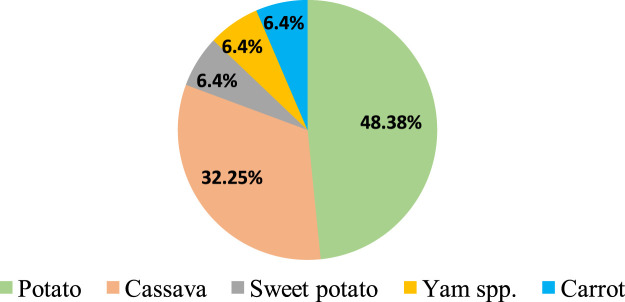
Crop-wise percentage of CRISPR/Cas gene editing reported in root and tuber crops.

Feasibility of CRISPR/Cas9-mediated genome editing in RTCs has been successfully demonstrated by targeted mutagenesis of *auxin/indole-3-acetic acid* family gene (*StIAA2*) (Wang et al., 2015) and *acetolactate synthase1* (*StALS1*) gene (Butler et al., 2015) in potato and *phytoene desaturase* in cassava (*MePDS*) (Odipio et al., 2017) and *Dioscorea rotundata* (*DrPDS*) (Syombua et al., 2021). Subsequently, CRISPR/Cas9 was deployed to improve different attributes of RTCs, and successful improvements were achieved in biotic stress resistance, herbicide resistance, starch profile modification, alleviation of toxicity, biofortification, and circumventing the barriers in sexual reproduction. In any case, genome editing has been reported only in five RTCs, of which majority reports (48%) are from potato (Figure 1). There are fewer reports regarding the application of gene editing to address aspects such as storability and abiotic stress resistance, post-harvest physiological deterioration, reprogramming of developmental patterns and metabolism for improved traits, improvement of root system architecture, and biofuel-oriented genomic modifications. A brief account of the significance of the aforesaid aspects, possible genetic manipulations using CRISPR/Cas to obtain a favorable impact, and putative target genes deciphered from previous gene functional studies which provide useful hints to channel future research in this field are also included in this review.

### 4.1 Biotic stress resistance

Biotic stress factors such as viral, fungal, bacterial, and nematode pathogens and different insect pests severely affect the production of RTCs.

Major pathogens infecting sweet potato are *Sweet potato feathery mottle virus* (SPFMV) and *Sweet potato leaf curl virus* (SPLCV), and the most destructive pest is the sweet potato weevil. *Alternaria solani* and *Phytophthora infestans* are the fungal pathogens causing early blight and late blight in potato, respectively. In addition to different fungal and viral pathogens, root-knot nematode, root-lesion nematode, and weevils are major biotic threats to sweet potato cultivation. Potato (*S. tuberosum* L.) is also affected by bacterial diseases like bacterial wilt and scab caused by *Ralstonia solanacearum* and *Streptomyces* sp., respectively, and viruses like *Potato leafroll virus* (PLRV) and *Potato virus Y* (PVY). Cassava mosaic virus is the major pathogen infecting cassava and causes cassava mosaic disease (CMD), resulting in a heavy yield loss. Major insect pests of cassava are cassava mosaic virus vectors, namely, white fly (*Bemisia tabaci*), and cassava mealybug (*Phenacoccus manihoti*) (Allemann et al., 2004). Other diseases affecting cassava are cassava brown streak disease (CBSD) and bacterial blight caused by *Xanthomonas axonopodis* pv*. manihotis*. *Dasheen mosaic virus* and fungal pathogen *P. infestans* are the major pathogens affecting *Amorphophallus*. The lack of disease-free, quality planting material is a prominent limiting factor in the cultivation of vegetatively propagated RTCs (Allemann et al., 2004). Even though conventional breeding programs came up with some resistant RTC varieties and transgenic techniques like RNAi could develop resistance to pathogens in certain RTC species, issues regarding efficiency, durability, and broad-spectrum resistance insist on the development of robust resistance mechanisms against pathogens infecting RTCs. This is particularly important due to the emergence of more virulent pathogens and combined infections under field conditions. Additionally, transgenic techniques for efficient control of major bacterial, fungal, and nematode pathogens infecting RTCs remain underdeveloped and require extensive research. Thus, genome editing can be a potential alternative to stimulate research in this regard by providing the opportunity for functional annotation of genes and targeted mutagenesis.

Previous studies have shown that post-transcriptional gene silencing (PTGS) of the *AC1* (Vanderschuren et al., 2007), *Rep* (*AC1*), *TrAP* (*AC2*), and *REn* (*AC3*) (Zhang et al., 2005) of *African cassava mosaic virus* (ACMV) and *AV1* and *AV2* of *Sri Lankan cassava mosaic virus* (SLCMV) (Ntui et al., 2015) confer cassava with resistance to ACMV and SLCMV, respectively. In addition to *Geminivirus* resistance, resistance to pathogenic *Ipomovirus*, namely, *cassava brown streak virus* (CBSV) and *Ugandan cassava brown streak virus* (UCBSV), which cause cassava brown streak disease (CBSD), was developed by PTGS of coat protein (*CP*) gene (Patil et al., 2011; Yadav et al., 2011). The above-described viral genes that were the targets for PTGS can be potential targets to be knocked out by CRISPR/Cas9, for developing virus resistance. Since CRISPR/Cas9-induced mutations are highly specific and stable, complications like the loss of natural *CMD2* resistance in certain transgenic cassava varieties with RNAi-mediated engineered resistance against CBSV over generations (Beyene et al., 2016) may be avoided. Transgenic approaches targeting conserved genomic regions of pathogens would be more efficient for conferring broad-spectrum resistance that is particularly advantageous in the case of multiple infections in the field. However, this could not impart complete resistance despite the delayed infection with mild symptoms exhibited by transgenic plants. An efficient alternative strategy for achieving broad-spectrum resistance can be the simultaneous targeting of multiple genomic regions of different viruses through multiplex editing facilitated by CRISPR/Cas9.

CRISPR/Cas9 may accelerate further investigation of the genetic basis of disease development, host–virus interaction, and integrated targeting of multiple genetic/molecular regulatory pathways of the infection process in different RTCs for imparting them with disease resistance. CRISPR/Cas9-induced loss-of-function mutations can facilitate the functional annotation of genes associated with disease resistance and susceptibility. Overexpression or activation of identified resistance genes and knocking out of host susceptibility genes is possible with CRISPR/Cas9 to enhance disease resistance. This tool can also be deployed for knocking out the integrated genome of pathogenic viruses like *Yam badnavirus*. Optimization of conditions to activate the HDR repair pathway upon CRISPR/Cas9- or CRISPR/Cpf1-induced DSBs may enable the incorporation of well-characterized resistance genes from resistant wild cultivars into farmer-preferred landraces.

The possibility of the evolution of Cas9 cleavage-resistant mutant virulent strains/isolates of pathogens is an inherent risk in targeting pathogenic genes. This is particularly important in the case of viral pathogens due to the high mutation rate of their genome. Mehta et al. (2019) encountered such a situation while targeting the multifunctional transcription activator (TrAP) protein-encoding *AC2* gene and the replication enhancer (REn) protein-encoding *AC3* gene of the *A. cassava mosaic virus*. They observed the evolution of edited viral genomes with a single nucleotide mutation that confers them with Cas9 cleavage resistance. Targeted editing generated premature stop codons in *AC2* and *AC3* and reduced the length of AC2 protein from 136aa to 62aa.

The newly evolved virus variant, namely, *ACMV-AC2 H54Q* possessed a single T insertion in H54Q of the *AC2* sequence. This mutation is responsible for premature stop codon and also created a new open reading frame (ORF), which coded for the missing amino acids of *AC2*. Despite the mutation, the *AC2* function is kept unaffected through a selected insertion mutation.

Interestingly, the inserted T is in the guide RNA seed sequence, and the insertion is selected during editing to confer Cas9 cleavage resistance. Thus, editing failed to confer ACMV resistance and the edited cassava lines exhibited no reduction in disease incidence, symptom severity, and viral titer. Simultaneous targeting of multiple genomic regions using CRISPR/Cas9-tRNA TU or deployment of paired Cas9 nickase for inducing large deletions may render the emergence of a fully functional pathogen impossible once it is subjected to Cas9 cleavage. Similarly, the mutation in one target region alone becomes insufficient to escape the pathogen genome from CRISPR/Cas9 action as more than one sequence is targeted in multiplexing. This is expected to be an efficient strategy to overcome the barrier created by the evolution of CRISPR/Cas9 recognition and cleavage-resistant pathogens to a large extent.

Certain host translation initiation factors function as host susceptibility factors and are potential targets to confer resistance to some RNA viruses. Mutation of a specific translation initiation factor, eIF4E, using CRISPR/Cas9 has conferred virus resistance in some crops. Gomez et al. (2019) successfully conferred tolerance to cassava brown streak disease by editing susceptibility genes in cassava. CBSD is mainly caused by *Cassava brown streak virus* and *Ugandan cassava brown streak virus* (UCBSV), both belonging to the genus *Ipomovirus* and family *Potyviridae*. Potyviral virulence factor, viral genome-linked protein (VPg) usually interacts with isoforms of eukaryotic translation initiation factors for its translation and subsequently establishes infection and the latter thus acts as a susceptibility factor. CBSV and UCBSV interact with two eIF4E isoforms, namely, novel cap-binding protein1 (nCBP-1) and nCBP-2 to cause infection. CRISPR/Cas9-edited cassava lines exhibited attenuated aerial symptoms and reduced severity of root necrosis which can be correlated with significantly reduced viral titer in them. Eukaryotic translation initiation factors could be a potential editing target in yam to impart resistance to *Yam mosaic virus* (YMV), *Yam mild mosaic virus* (YMMV), *Yam asymptomatic virus 1* (YaV1), and *Dioscorea mosaic-associated virus* (DMaV) (Syombua et al., 2022).

Sweet potato virus disease (SPVD) is the most important biotic stress affecting sweet potato production worldwide. Synergistic interaction of a *Potyvirus* and *Crinivirus*, namely, *Sweet potato feathery mottle virus* (SPFMV) and *Sweet potato chlorotic stunt virus* (SPCSV), respectively, cause severe SPVD resulting in a yield loss of up to 90%. Recently, Yu et al. (2022) could successfully confer resistance to SPCSV which induces virus synergism along with SPFMV to cause SPVD. SPCSV-encoded dsRNA-specific class 1 RNase III endoribonuclease (RNase3) is an important pathogenicity factor that suppresses the host’s major antiviral defense mechanism, post-transcriptional gene silencing (PTGS), by the processing of 21 nt viral siRNAs to 14 nt siRNAs and thereby facilitating infection. Targeted editing of SPCSV *RNase3* with two different RNA targeting CRISPR/Cas13 orthologs of subtypes a and d namely, *L. wadei* Cas13a (LwaCas13a), and *Ruminococcus flavefaciens* Cas13d (RfxCas13d) imparted significantly enhanced resistance to SPVD by restoring host PTGS against the virus. Further optimization of gRNA expression promoters and identification of potential targets in both SPFMV and SPCSV would be useful to confer robust SPVD resistance in sweet potatoes.

Zhan et al. (2019) employed RNA targeting CRISPR/Cas13a for conferring resistance against *Potato virus Y*. Targeted editing of genomic sites of *P3*, *CI*, *NIb*, or *CP* that are conserved across PVY strains resulted in resistance specifically against the three strains, namely, PVY^O^, PVY^N^, and PVY^N:O^. This study also evidenced that the degree of resistance imparted by editing is correlated to the expression levels of Cas13a and sgRNAs.

Recently, RNA targeting *R. flavefaciens* CRISPR/Cas13d system was employed to confer broad-spectrum resistance against four single-stranded RNA viruses infecting potato, namely, *Potato leafroll virus* (PLRV), *Potato virus Y* (PVY), *Potato virus X* (PVX), and *Potato virus S* (PVS), through multiplex editing (Zhan et al., 2023). Four guide RNAs targeting the *CP* region of the four viruses were assembled to create a polycistronic tRNA-gRNA gene, and this construct was used for transformation. Transgenic plants regenerated after *Agrobacterium*-mediated transformation expressed four gRNAs, and challenge inoculation revealed the potential of the PTG CRISPR/Cas13d system to provide resistance to multiple viral infections. All transgenic potato lines exhibited a significant reduction in virus accumulation and the obvious symptom was absent. Class VI effector Cas13d is comparatively small and hence is suitable for viral vector-mediated delivery of CRISPR/Cas components as well.

Coilin is a nucleolar protein localized in the Cajal bodies of the nucleolus, known for its role in plant defense against virus infections, particularly implicated through its interaction with the salicylic acid pathway and signaling. Makhotenko et al. (2019) devised a *Potato virus Y* resistance, conferring the CRISPR/Cas9 system by editing the coilin gene in potato cultivar Chicago. Targeted editing of the C-terminal domain (CTD) of the coilin gene was done by particle bombardment of Cas9-gRNA RNP complex into the apical meristem, and a significant increase in resistance to PVY was achieved. In addition, coilin gene editing also conferred remarkable resistance to salt and osmotic stress. Interestingly, editing of a single allele provides potato plants with resistance to PVY and abiotic stress while other alleles remain switched on and the coilin expression is not completely inhibited, thereby rendering it a promising editing target for multiple trait improvement in potato. The impact of coilin gene editing on other RTCs has to be evaluated.

Bacterial blight (BB) caused by *X. axonopodis* pv*. manihotis* (*Xam*) is an important threat to cassava cultivation as it results in devastating damage to crop yield, next to CMD. In susceptible varieties, *Xam* alters the expressions of *SWEET* family genes that regulate sugar translocation. Transcription activator-like 20 (TAL20) effector of *Xanthomonas* binds to the effector binding elements (EBE) in the promoter of the *MeSWEET10a* gene and induces its overexpression to redirect sugar flux toward the infection site. CRISPR/Cas9-mediated editing of TAL20 binding site in the *MeSWEET10a* EBE in cassava resulted in repressed *MeSWEET* expression and increased callose deposition in cell walls which in turn conferred resistance to cassava bacterial blight (Wang et al., 2022). The same target was edited by Elliott et al. (2023) using a dual gRNA system against *Xanthomonas phaseoli* pv. *manihotis* (*Xpm*). They used four different combinations of five gRNAs targeting TAL20 EBE, translation start site, *MeSWEET10a* 5-′UTR upstream of the start codon, upstream of the TATA box, and TAL20 EBE downstream of the TATA box. All produced mutations in the *MeSWEET10a* promoter or coding region, and this in turn conferred resistance to BB, evidenced by highly reduced lesions characteristic of *Xpm*. They also found that despite its localized expression in flower tissue, the mutations in *MeSWEET10a* are not affecting flowering and reproductive function and hence prove this to be suitable for field application. *IbSWEET10* also could be a suitable target to manipulate in the same way to impart resistance to *Fusarium oxysporum* in sweet potatoes (Li et al., 2017). Activation of the genes *DELLA*, *bZIP*, *RAV1*, and *RAV2*, which are involved in defense response pathways, are effective in conferring resistance to bacterial blight (Wei et al., 2018). So, CRISPRa-induced activation of these genes indeed would be an efficient strategy to impart BB resistance in cassava.

Targeted editing of host susceptibility genes confers resistance to late blight, one of the most dreadful diseases of potato (Kieu et al., 2021). Late blight disease is caused by an oomycete pathogen *P. infestans*. Seven candidate susceptible genes, namely, *MLO1* (*Mildew locus O*), *HDS* gene homolog, *AtTTM2* gene homolog, *StDND1*, *StCHL1*, and other two *DMR6* homologs were targeted using dual gRNA strategy. Out of the seven candidates, tetra allelic mutations in three genes, *StDND1*, *StCHL1*, and *StDMR6-1*, were found to impart the resistance. However, mutations in *StDND1* harm the phenotype and hence is not a suitable candidate for field applications. Contrary to this, *StCHL1* and *StDMR6-1* mutants had no significant difference in growth and phenotype. In addition, the latter remarkably enhanced the resistance to the pathogen.

In a recent study, Bi et al. (2023) identified a novel susceptibility factor involved in the defense response to *P. infestans*, namely, *S. tuberosum plasma membrane protein 1* (*StPM1*), encoded by *ABA-induced wheat plasma membrane polypeptide-19* (*AWPM-19*)–like family gene. CRISPR/Cas9 mediated knockout of *StPM1* resulted in a considerable reduction of disease symptoms without hampering the growth and development of the plant. In addition, *StPM1* knockout mutants exhibited upregulated expression of defense-related genes like *StPR1*, *StPR5*, *StWRKY7*, and *StWRKY8* (Bi et al., 2023). This study suggests *StPM1* as a potential target for editing to confer potato with resistance to *P. infestans.* The identification of other suitable editing targets in the potato genome to obtain resistance to late blight pathogen can have promising impacts since the extent of yield loss caused by this disease is high.

Precise insertion of anti-fungal peptide into the RTC of interest is also possible with CRISPR/Cas9-induced HDR. Thus, it would be worthy to characterize genes encoding anti-fungal peptides in RTC species and their appropriate manipulation using CRISPR/Cas9 to improve resistance to fungal pathogens. Fan et al. (2015a) have shown that silencing of *unc-15* gene encoding paramyosin, a protein related to muscle movement, is useful to control sweet potato-infecting nematode, *Ditylenchus destructor.* CRISPR/Cas9-mediated silencing strategies can be employed for controlling pathogenic nematode infecting RTCs by targeting similar candidate genes regulating muscle movement.

Since insect vectors play an important role in the transmission of a pathogenic virus, efficient strategies to control them are highly significant. Gene editing techniques are useful for developing insect and pest resistance as well. An insecticidal property of sporamin in sweet potato, which is attributed to its trypsin inhibitory activities, has been evaluated in previous studies (Senthilkumar and Yeh, 2012). The key transcription factors (TFs) determining sporamin expression and wounding response, namely, NAC domain protein (*IbNAC*) and *IbWRKY1* can be appropriate candidates for editing to upregulate sporamin expression so that protection against mechanical wounding and herbivory can be enhanced (Chen et al., 2006; Chen et al., 2016). Previous studies have found that *IbNAC*- and *IbWRKY1-*mediated sporamin expression is jasmonic acid (JA) and salicylic acid (SA) pathways dependent. So, the binding of two other TFs *MYC2/4* and *JAZ2/TIFY10A* (jasmonate ZIM/TIFY-domain) that regulate JA and SA signaling also can be modified either through inducing mutations in their binding sequences or within their binding domains using CRISPR/dCas9 (Rajendran et al., 2014). In addition to sporamin, taro cystatin and chitinase have the potential to confer broad-spectrum resistance against insect pests (*Spodoptera litura* and *Spodoptera exigua*), pathogens (*Alternaria alternata* and *Pectobacterium carotovorum* subsp. *carotovorum*), and osmotic stress in heterologous species (Chen et al., 2014). Thus, CRISPR/Cas9-mediated gene insertion strategies may be applied for stacking such genes in RTC species. Chitinases, lipoxygenases, caffeoyl-CoA-o-methyltransferase, and LOX5 can be potential targets in cassava for developing resistance against white flies (Chavarriaga-Aguirre et al., 2016).

Modification of volatile chemicals present in RTCs that have insect-deterring properties may be possible with CRISPR/Cas9 to make plants insect resistant. (E)-β-farnesene (Eβf) is a volatile hydrocarbon released by aphid-infested plants, and this attracts the parasitic wasp *Diaeretiella rapae*. Subsequently, the wasp feeds on the aphids and thereby reduces the aphid population (Tyagi et al., 2020). Host plant genes encoding such compounds could be manipulated by CRISPR/Cas9 for efficient pest control. Another way is the alteration of plant appearance by editing respective pigment biosynthetic pathways, which would render them non-identifiable by pests (Malone et al., 2009). Knocking out of the cadherin receptors in insect midgut, which act against insecticidal proteins (Wang et al., 2016), and modifying the pest detoxification genes, like the gossypol-inducing cytochrome P450 genes (Tyagi et al., 2020), by CRISPR/Cas9 will increase the susceptibility of insects to insecticides so that the mutant insects can be easily targeted using insecticides. Interruption of chemical communication for various purposes among pest populations by targeting the olfactory receptors of insects/pests could control pest populations (Wang et al., 2016). Pest developmental genes are another suitable target for editing. This is demonstrated against fall armyworm (FAW) *Spodoptera frugiperda* (J.E. Smith) by targeted editing of the *abdominal-A* (*Sfabd-A*) gene wherein reproductive development of the insect is impaired and thereby enables FAW population control (Wu et al., 2018). Using CRISPR/Cas9 genome editing technique to confer RTCs with efficient and durable resistance against major pests and pathogens would not only reduce the associated crop loss but also save the expenses on pesticides, insecticides, other disease management measures, and labor charges.

### 4.2 Herbicide tolerance

Massive weed infestation is a serious constraint in the production of RTCs. Although the availability of herbicides has avoided the requirement for labor-intensive and time-consuming manual weeding, certain herbicides have been found to cause some adverse effects on crop plant growth and development in varying degrees (Enyong et al., 2013; Ekeleme et al., 2020). CRISPR/Cas9 can be employed for precise insertion of herbicide tolerance conferring genes like the *5-enolpyruvylshikimate-3-phosphate synthase* (*EPSPS*) or *phosphinothricin acetyltransferase* (*bar*) gene in RTC species. This has been done in cassava for glyphosate resistance by Hummel et al. (2018). They replaced the cassava native promoter for *EPSPS* with a constitutive promoter by CRISPR/Cas9 editing-induced homologous recombination. Editing with gRNAs targeting *EPSPS* promoter and second intron created 3.2 kb deletion. The disrupted region was replaced with a repair template comprising a strong constitutively expressed promoter by homologous recombination. Double amino acid substitutions were also introduced by NHEJ, and this provided fitness to the plant while the native promoter was fully replaced. Thus, promoter swapping and amino acid substitution by CRISPR/Cas9-induced HR and NHEJ, respectively, conferred glyphosate resistance in cassava.

Butler et al. (2015) and Butler et al. (2016) developed herbicide-resistant potato by editing the *acetolactate synthase* (*ALS1*) gene using both CRISPR/Cas9 and TALEN. In this study, two potato cultivars (tetraploid and diploid) were subjected to the editing of the two codons corresponding to W563 and S642 in the 3′ coding region of the *ALS1* gene. They used a BeYDV-derived geminivirus replicon (GVR) vector harboring a T-DNA with *ALS1* repair template and SSNs (CRISPR/Cas9 and TALEN) flanked by short intergenic region (SIR) and long intergenic region (LIR) and another vector expressing the *Rep* gene. The repair template with amino acid substitution conferred herbicide-tolerant phenotype, while the essential functions of *ALS1* in amino acid biosynthesis were retained. Edited lines exhibited reduced susceptibility to herbicide imidazolinone and improved growth. In addition, this study provided successful validation of two aspects, the effectiveness and utility of GVR vectors for plant gene editing, particularly to promote HR, and the enhancement of gene targeting modifications induced by regeneration of secondary events under high selection pressure.

The development of a transgene-free, chlorsulfuron-resistant potato by employing a cytidine base editor (CBE) is another remarkable achievement. The CBE construct was designed to target the region covering proline 186 of *StALS*, and out of the 75% mutated lines, 10% was found to be transgene free after screening.

### 4.3 Modification of starch profiles of storage root/tuber

The culinary qualities of storage tubers and roots can be improved by modifying the structural and functional characteristics of biochemical components by editing the underlying genetic elements, and this will considerably increase their demand. Physicochemical modifications of biochemical and structural components of storage tubers can improve their compatibility for making value-added products for food and industrial purposes, which will broaden their utility and profitability. Usually, physical and chemical methods or enzyme-mediated processes are used for such industry-oriented starch modification and are expensive and laborious. CRISPR/Cas9 can be used to induce precise mutations in genes involved in starch biosynthesis. This enables the modification of quantity, quality, physical–chemical–structural properties of starch that determine its cooking/baking quality, taste, gel consistency, clarity, fermentability, gelatinization, retrogradation, rheological properties, and digestibility specific to the end product. This would benefit industries by eliminating the requirement for modification of starch which in turn reduces the expense and time incurred in the respective processes.

Tubers with customized novel starch profiles enhance their utility for specific purposes like food, biofuel, livestock feed, and industries like textile, pharmaceutical, and paper manufacturing. The candidate targets that can be modified are the key enzymes involved in starch biosynthesis, for example, ADP-glucose pyrophosphorylase and granule-bound starch synthases (GBSS) driving amylose biosynthesis and soluble starch synthases (SSS), starch branching enzyme (SBE or BE) and debranching enzyme (DBE), which are involved in amylopectin synthesis in plants such as cassava (Zhou et al., 2020). Previous studies have shown that silencing of the *granule-bound starch synthase1* (*GBSSI*) gene in cassava by RNAi techniques yield low amylose starch with features preferred for applications in paper, textile, and food industries. Since this novel starch has improved stability and clarity and the smooth and shiny texture of gels, the adverse effects of retrogradation on the functionality of starch, which is primarily attributed to amylose content can be avoided. This eliminates the requirement for expensive and complex chemical treatment or processing as well.

Considering *GBSSI* as a target for editing in cassava to obtain amylose-free starch with improved functionalities which is superior to unmodified (amylose containing) cassava starch and waxy cereal starch (Jobling, 2004; Raemakers et al., 2005; Zhao et al., 2011; Koehorst-van Putten et al., 2012), Bull et al. (2018) edited *MeGBSSI* using CRISPR/Cas9. Phenotypic characteristics and plant growth remained intact in edited lines and no off-target editing was observed. Edited lines showed modified pasting and gelatinization properties that are more desirable for various industrial purposes. Bull et al. (2018) considered *protein targeting to starch* (*PTST1*) gene as the target for editing in cassava as its important role in the localization of GBSSI on starch granules in the chloroplast is known from a previous study in *Arabidopsis* by Seung et al. (2015). They successfully created low amylose-containing starch by CRISPR/Cas9-mediated targeted mutagenesis of *MePTST1*. A 40% reduction in amylose content was obtained. Additionally, the flowering of edited plants was accelerated by transgenic expression of the flowering-inducing gene *FLOWERING LOCUS T of Arabidopsis* (*AtFT*) so that successive non-transgenic edited lines could be segregated and evaluated within less time, unlike the several year-consuming processes of conventional breeding.

Amylose-free starch-synthesizing potato is also created by targeted knockout of the *GBSSI*. Exon 4 of *GBSSI* was subjected to CRISPR/Cas9 RNP-mediated protoplast transformation. Andersson et al. (2018) employed two different RNPs, one with transcribed gRNA and the other with synthetic gRNA, and both RNPs produced knockout mutants with a mutation in all four alleles and free of any unwanted DNA inserts with a knockout success rate of 2%–3%. This study provided more insight into the efficiency of different RNP-mediated transgene-free editing in potato for trait improvement.

The same gene was edited by Veillet et al. (2019) using a modified strategy wherein two gRNAs targeting exon 1 and exon 2 were used to edit *GBSSI* through *Agrobacterium-*mediated and protoplast transformation methods. Even though both resulted in successful editing of the targets, there were certain drawbacks like the presence of residual transgene in the protoplast-regenerated plants, random foreign DNA insertions, the possibility of somaclonal variation of plants regenerated after transformation, and abnormal growth or development.

However, the efficiency of this editing strategy in different potato cultivars was confirmed in this study by applying the same in another cultivar, Furia, with the recovery of 19% tetra-allelic mutations following the transformation. In addition to this, base editing to target two catalytic active domains of *GBSSI*, encoding KTGGL and the PSRFEPCGL on exon 1 and exon 10, respectively, with cytidine base editors (CBE) was also done. Base substitution from C-17-to-G-17 in the KTGGL region was observed, and this caused the impairment of *StGBSSI*.

Class A starch-branching enzyme-encoding gene in sweet potato, *starch-branching enzyme* (*IbSBEII*), is a suitable candidate for modification as its silencing has been found to produce amylase-rich starch (Shimada et al., 2006; Lyu et al., 2021). In sweet potato, targeted mutagenesis of *IbGBSSI* and *IbSBEII* was done to modify the starch profile (Wang et al., 2019). The efficiency of single and dual gRNA systems was evaluated in this study. For *GBSSI*, a single and dual gRNA system was designed to target the first exon, while for *IbSBEII*, a single gRNA targeted exon 15 and a dual gRNA system targeted exon 12 and exon 15. A mutation efficiency of 62%–92% was observed, and a dual gRNA system was found to be more efficient than single gRNA in creating mutations. The amylose content was increased in *IbSBEII* knockout mutants, whereas low amylose starch was produced by *GBSSI* knockout mutants. A recently developed cut–dip–budding transformation system was successfully employed for editing *IbGBSSI*, *IbSBEI*, and *IbSBEII*. Edited plants were developed from transgenic roots and possessed starch with intended modifications. The efficacy of this transformation system for gene editing in multiple cultivars has been confirmed by editing the *phytoene desaturase* (*PDS*) gene (Cao et al., 2023).

Genes involved in phosphorylation or dephosphorylation of starch such as *glucan water dikinase* (*GWD*), *starch excess 4* (*MeSEX4*), and *like sex4 2* (*MeLSF*) can be appropriately modified using CRISPR/Cas9 for improving certain functional properties influenced by phosphate content in starch, like swelling power and paste clarity (Wang et al., 2018). The *starch-binding domain* (*SBD2*) is another suitable candidate to be modified for obtaining improved granule morphology while the primary structure of the constituent starch molecules remains unaltered (Zhang et al., 2013).

Apart from starch remodeling, CRISPR/Cas9-induced targeted mutagenesis can bring about the downregulation of genes involved in polyphenol production, such as polyphenol oxidase, in sweet potato tubers. This enhances the value of sweet potato tubers in the food industry as low polyphenol content is preferred for making flour-based chips (Joint, 1987). Designer crops with engineered components or metabolic pathways, like those achieved through CRISPR technology improve their suitability for multiple purposes and reduce the expense, complexity, and pollution associated with conventional processing methods.

### 4.4 Alleviating toxicity/anti-nutritional contents

RTCs have the presence of certain toxic or anti-nutritional components like alkaloids and glycosides that require time and energy-consuming processing before usage.

The presence of cyanogenic glycosides is an important undesirable trait of cassava. Cyanogenic compounds will release toxic cyanide upon cellular disruption. This will interfere with cellular respiration and eventually cause cell death. Cyanide poisoning due to improper processing and chronic cyanide intake through diet affects the nervous system and can even be fatal. Principal cyanogens in cassava are linamarin and lotaustralin. Cyanide-free cassava is a prime objective of cassava improvement programs. Cyanide levels in cassava get elevated under drought stress. So, cassava-consuming communities experiencing severe incidents of drought are exposed to the risk of high cyanide intake. The heavy dependence on a cassava diet with low protein consumption also leads to cyanide toxicity. Acyanogenic cassava provides safe staple food for cassava-consuming communities. In addition to this, there are advantages like the elimination of the requirement for labor-intensive and time-consuming processing for detoxification of harvested tubers, the prevention of water pollution that occurs during processing, and expense and labor required for purifying the water used for detoxification.

CRISPR/Cas9 can be applied to knock out the key genes or regulatory elements involved in cyanoglucoside biosynthesis. Two paralogous genes *CYP79D1* and *CYP79D2*, catalyze the rate-limiting step of cyanogen biosynthesis (Andersen et al., 2000) and have been found as ideal targets for manipulation to reduce cyanogen content (Siritunga and Sayre, 2004). Precise editing of *CYP79D1* and *CYP79D2* with a dual guide RNA CRISPR/Cas9 system resulted in cyanide-free cassava without the need for permanent transgene expression (Gomez et al., 2023). Knockout of *MeCYP73D2* alone is sufficient to inhibit cyanide biosynthesis, whereas *CYP79D1* knockout did not yield the desired result. However, Juma et al. (2022) demonstrated that CRISPR/Cas9-induced *CYP79D1* knockout mutants also are cyanide free. In this case, the homology of the targeted regions in *CYP79D1* to that of *CYP79D2* indicates the possibility that editing occurred in the respective regions of *CYP79D2* instead of *CYP79D1* (Bredeson et al., 2021). Since dual knockout plants were morphologically similar to the wild type, it is obvious that the inhibition of cyanide biosynthesis has no significant implications on nitrogen metabolism and plant growth. The successful assessment of the consistency of the same CRISPR/Cas9 dual knockout system for conferring the desired phenotype across three cultivars, 60444, TME 419, and an improved variety TMS 91/02324, suggests that this is a promising strategy.

Siritunga and Sayre (2004) and Jorgensen et al. (2005) have suggested that knockout of the cassava ortholog of *UDP-glycosyl transferase*, which is involved in the conversion of linamarin to linustatin, can render tubers acynogenic. Enhancing the expression of *hydroxynitrile lyase* (*HNL*) in tuberous roots using root-specific expression of CRISPR/Cas9 cassettes can be a potential strategy to reduce cyanogenic content in roots (Siritunga et al., 2004).

Steroidal glycoalkaloids (SGA) like α-solanine and α-chaconine present in potato tubers cause bitter taste and are toxic to certain organisms. Elimination or reduction of SGAs in tubers is a prime goal in potato improvement programs. Certain RNAi approaches can reduce SGA content by silencing SGA biosynthesis genes but do not achieve complete elimination. SGA-free potato tuber was developed by Nakayasu et al. (2018) through CRISPR/Cas9-mediated editing of a gene involved in SGA biosynthesis, namely, *St16DOX*, encoding 16α-hydroxylase. Multiplex editing of *St16DOX* in a hairy root transformation system resulted in the elimination of SGAs while there was an accumulation of glycosides of 22,26-dihydroxycholesterol, the substrate for SGAs. However, this is a transient system that can be used for the evaluation of more targets. Evaluated genes must be targeted in a stably transformable and heritable system for further applications.

CRISPR/Cas9 can be deployed for modification of biosynthetic pathways for directing the intermediate products into the harmless or comparatively less harmful or beneficial compound, either by alteration of enzymatic activities or by the introduction of novel genes encoding certain enzymes or competing substrates that can interfere in the pathway and drive it in a desirably modified direction. Trypsin inhibitors in sweet potato which impede the availability of proteins from tubers and alkaloids and tannins present in some yam species are non-preferable characteristics. Their genetic regulatory components can be considered suitable targets for editing (Lebot, 2010). Editing of genes regulating calcium oxalate metabolism, its deposition in tissues, and raphide formation may help get rid of the acridity of tubers.

One of the most predominant yam species in Africa and West Africa, *Dioscorea dumetorum* possesses desirable attributes like high nutritional value, a remarkably good balance of essential amino acids, high yield (40 t/ha), and minimum labor-intensiveness. Yet, this is the least cultivated one due to an undesirable phenomenon called post-harvest hardening. A gene functional analysis study by Siadjeu et al. (2021) found that post-harvest hardening can be attributed to the upregulation of five genes, *MYB* transcription factor, *chlorophyll a/b-binding protein1*, *2*, *3*, *4* (*LHCB1*, *LHCB2*, *LHCB3*, and *LCH4*), *xylan o-acetyltransferase* (*XOAT*), and *cellulose synthase* (*CESA*). CRISPR/Cas9 mediated multiplex editing strategies could be deployed for the silencing or to downregulate the expression of these genes so that post-harvest hardening of *D. dumetorum* tubers can be minimized.

### 4.5 Breaking barriers in RTC breeding: CRISPR/Cas9 for breaking self-incompatibility and flowering induction

Introgression of desirable genes through breeding is complicated in commonly cultivated potato varieties due to their polyploid nature. So, re-domestication of potato as a diploid inbred/F1 hybrid variety is a preferred alternative for producing new varieties comprising favorable allelic combinations and elite phenotypic attributes. However, self-incompatibility governed by a single multiallelic S-locus is a major constraint in breeding diploid potatoes. S-locus comprises pistil-expressed S-locus RNase (*S-RNase*) and the pollen-expressed F-box proteins (*S locus F-box* or *SLF*). The former has a cytotoxic effect on self-pollen, whereas the latter forms a part of a detoxification complex that allows compatible non-self-pollen to enter the style. CRISPR/Cas9 was employed to overcome the self-incompatibility by using a dual sgRNA system targeting the first two exons of *S-RNase*. Premature stop codons were generated by multiple biallelic and homozygous deletions in the target genomic site. This hampered the expression of *S-RNase* in mutant T0 and T1 lines, which in turn resulted in self-compatibility (SC) (Enciso-Rodriguez et al., 2019). Successful induction and stable inheritance of SC conferring mutations is a promising step in the attempts to develop diploid inbred potato varieties.

Genome editing techniques can augment the constraints in cassava breeding programs like low rate of natural fertility and delayed and non-synchronous flowering. Precise manipulation of expression patterns of genes regulating cassava flowering in a spatiotemporally regulated manner for generating flowering induction signals can be possible with CRISPR/Cas9 editing tools. This may result in early and profusely flowering edited lines that would serve as the parental population for the crossing, which in turn come out with elite hybrids. Activation and repression of enhancers and inhibitors of flowering, respectively, are possible with the CRISPR/dCas9 tool. Previous studies have suggested the *phosphatidylethanolamine-binding protein* (*PEBP*) family of genes, *FLOWERING LOCUS T* (*FT*) gene orthologs *MeFT1* and *MeFT2*, and *TERMINAL FLOWER1* (*TFL1*) gene as suitable candidate targets in this respect (Adeyemo et al., 2017 and Adeyemo et al., 2019). This is evidenced by the induction of accelerated flowering by CRISPR/Cas9-mediated expression of *Arabidopsis FLOWERING LOCUS T* GENE (*AtFT*) in cassava (Bull et al., 2018). Furthermore, Odipio et al. (2020) reported the flowering induction in cassava within a short period of 21 days by editing a native gene, namely, *MeFT1*. The regulatory role of *MeFT1* was evidenced by the upregulation of downstream floral meristem identity genes *MeAP1*, *MeSOC1*, and *MeLFY* upon its overexpression. The elevated expression of floral meristem identity genes contributed to early flowering. So, they can be considered candidate targets for editing in the future. This strategy is valuable for accelerating cassava breeding, which typically requires approximately 6 years with a natural flowering cycle (Bull et al., 2017; Bull et al., 2018). However, the constitutive overexpression of *FT1* adversely affects the number and weight of tubers, unlike in potato and onions. This indicates the necessity of devising an inducible *FT1* overexpression system with suitable promoters to obtain flowers for breeding without compromising the yield. Inducible CRISPR/Cas9 systems enable controlled editing and thereby mitigating the disadvantages associated to constitutive expression.

Low flowering rates under natural conditions, specific photoperiodic and temperature requirements, and complications due to sexual incompatibility are the hurdles in sweet potato breeding programs. Although there have been comparatively fewer studies regarding genetic and molecular factors regulating flowering in sweet potato, certain flowering-associated putative genes like *LEAFY* (*LFY*), *AGAMOUS* (*AG*), *TERMINAL FLOWER* (*TFL*), *FLOWERING LOCUS C* (*FLC*), *CONSTANS* (*CO*), *APETALA1* (*AP1)*, *APETALA2* (*AP2*), *APETALA3* (*AP3*), *DELLA*, and *SLEEPY1* (*SLY1*) have been identified in some previous studies (Samba, 2013; Tao et al., 2013). CRISPR/Cas9 can be employed for characterizing more genes that regulate flowering and for manipulating them appropriately to induce flowering. It can also be used to modify genetic or molecular factors behind incompatibility, which in turn can promote breeding programs. Genome editing strategies may complement the prevailing techniques used for inducing flowering in RTC species like grafting, photoperiod modulation, and the use of plant growth regulators.

### 4.6 Biofortification

Biofortification is one of the prime goals of RTC improvement programs, as malnutrition is a serious issue in regions where RTCs are the staple diet. The levels of certain essential nutrients and minerals are suboptimal in RTCs. CRISPR/Cas9-mediated editing can be applied to improve the protein content and amino acid profile either by modifying the expression of genes underlying the amino acid and/or protein synthesis or by precisely inserting the genes encoding the amino acids that are deficient in the RTC species. Cassava hydroxynitrile lyase (HNL) enzyme catalyzes the conversion of acetone cyanohydrin to cyanide and accelerates the root cyanogen metabolism for protein synthesis. Modification of *HNL* gene expression using CRISPR/Cas9 could be an efficient strategy for turning the root into a strong protein sink, thereby resulting in double advantage, elevated total root protein levels and reduced root cyanogen levels, so that the toxicity is alleviated. The effectiveness of this strategy is evident from a two- to three-fold increase in root protein and an 80% reduction in root cyanogenic content observed in transgenic cassava expressing *HNL* under root-specific patatin promoter (Siritunga et al., 2004; Sayre et al., 2011). Enrichment of tubers may be possible with the spatiotemporal modification of the expression of genes regulating biosynthesis and transport of nutrients/metabolites using the CRISPR/Cas9 construct designed with tissue- or developmental stage-specific promoter. Feasibility of using root/tuber specific promoters like *GBSSI* promoter, class I patatin promoter, p15/1.5 of a cytochrome P450 protein, and p54/1.0 of the cassava glutamic acid-rich protein, Pt2L4 for devising CRISPR/Cas9 cassettes for storage root-specific editing has to be evaluated. This will be useful for editing that is intended to upsurge the storage of carbohydrates (Zhang et al., 2003; Ihemere et al., 2006; Koehorst-van Putten et al., 2012).

The feasibility of applying gene editing-based strategies for the metabolite profile manipulation in RTCs has been evaluated in *Dioscorea zingiberensis*, wherein CRISPR/Cas9-induced mutation of the *farnesyl pyrophosphate synthase* (*FPS*) gene resulted in its reduced transcription, which in turn reflected in squalene content in the rhizome (Feng et al., 2018). *FPS* gene product catalyzes the condensation of dimethylallyl diphosphate (DMAPP) and geranyl diphosphate (GPP) to produce the Ɛ-isomer farnesyl pyrophosphate (FPP), one of the precursors of diosgenin. *D. zingiberensis* belongs to the Dioscoreaceae family and is well known for its high diosgenin content in the rhizome. Despite the enormous pharmacological potential and high demand for diosgenin, *D. zingiberensis* cultivation has decreased, and the low seed set rate is a barrier to its conventional breeding. The successful development of transformation and regeneration protocols for *D. zingiberensis* (Shi et al., 2012) and the recent establishment of the protocols for its CRISPR editing would augment the ongoing genetic engineering–based attempts to create *D. zingiberensis* with high diosgenin content.

CRISPR-mediated editing is effective to engineer the production of various secondary metabolites present in RTCs like dioscorin, diosgenin, sporamine, glycoalkaloids, phenolics, and saponins in a favorable direction. CRISPR/Cas9 and modified Cas9 forms can be employed for the transcriptional regulation of genes involved in the biosynthesis of secondary metabolites and to redirect their intrinsic metabolic flux, without affecting normal metabolism. This will enhance or reduce the production of the metabolite of interest. Targets of editing are determined depending upon the strategy to be used, i.e., whether to enhance or redirect the metabolic flux into the synthesis of a metabolite or to downregulate its catabolism. Candidate targets to promote beta carotene enrichment in RTCs can be the *lycopene epsilon-cyclase* (*LCYE*) gene, which directs the pathway toward the biosynthesis of ε-carotenoids or activating the expression of *lycopene beta-cyclase* (*LCYB*) gene, which catalyzes the formation of β-rings (Cunningham et al., 1996). *Phytoene synthase* and *1-deoxy-D-xylulose-5-phosphate synthase* (*DXS*) are other suitable candidates for editing, to achieve provitamin A fortification of RTCs, as their transgenic expression in cassava can produce higher provitamin A-containing tubers (Welsch et al., 2010). The key genes regulating the crucial steps of carotenoid and anthocyanin metabolism like *beta-carotene hydroxylase* (*CHY-beta*) (Kim et al., 2012), *geranylgeranyl pyrophosphate synthase* (*IbGGPS*) (Chen et al., 2015), *anthocyanidin synthase* (*IbANS*) (Welford et al., 2005), *MYB transcription factor* (*IbMYB1*) (Park et al., 2015), *dihydroflavonol-4-reductase* (*IbDFR*) (Wang et al., 2013), and *chalcone isomerase* (*IbCHI*) (Guo et al., 2015) can be potential targets to be edited for enrichment of sweet potato tubers with carotenoids and anthocyanins. This could confer health benefits such as antioxidant activities and salt stress tolerance.

### 4.7 Abiotic stress resistance

Drought affects yield and is negatively correlated with the nutritional quality of cassava tubers. Drought-affected tubers contain high levels of the cyanogenic glycoside (Vandegeer, et al., 2012). Although cassava and sweet potato are usually considered drought-tolerant species, the root and tuber yield of these crops are affected by extreme drought (Daryanto et al., 2016).

A study conducted by Ramirez-Gonzales et al. (2021) identified central clock output transcription factor CYCLING DOF FACTOR 1 (StCDF1) and its long non-coding RNA (lncRNA) counterpart, *StFLORE*, as two potential editing targets to regulate water homeostasis and vegetative reproduction in potato. StCDF1 together with StFLORE fine-tunes water homeostasis by regulating multiple factors like stomatal growth, size, density, and guard cell dynamics. *StFLORE* is also known to regulate ABA transcription and stomatal response to ABA signaling. CRISPR/Cas9 was employed to induce loss-of-function mutation of *StFLORE* by targeting four genomic regions that included the CDF1-binding site. *StFLORE* knockout mutants were more sensitive to water stress; however, the tuberization remained unaffected, whereas an overexpression of *StFLORE* under 35S promoter resulted in enhanced tolerance to water stress.

Hua et al. (2020) investigated the significance of the cyanide respiratory pathway in alleviating oxidative damage and regulating photosynthesis and photorespiratory reactions, especially under high light stress using CRISPR/Cas9. Targeted mutation of the mitochondrial alternative oxidase (*StAOX*) gene, an important component of the cyanide respiratory pathway, using CRISPR/Cas9 caused loss of *AOX* which in turn inhibited the cyanide respiratory pathway. This resulted in an increased accumulation of ROS, oxidative damage, increased membrane lipid peroxidation, reduced rate of photosynthesis, and increased levels of enzymes involved in photorespiration and malate–oxaloacetate shuttle route under high light conditions. This LOF analysis revealed the role of *StAOX* and associated cyanide respiratory pathways in abiotic stress management, photosynthesis, and carbon assimilatory pathways. From this study, we can consider the possibility of conferring plants with enhanced protection under high light stress by CRISPR-mediated transcriptional activation of *AOX* or other regulatory genes associated with it.

Precise modification of stress-responsive genes or genes encoding components of stress regulatory pathways and negative regulators of stress resistance genes is useful to improve stress tolerance in RTCs. Genes like *spermidine synthase* (*FSPD1*) (Kasukabe et al., 2006), *9-cis-epoxycarotenoid dioxygenase* (*NCED1*) (Estrada-Melo et al., 2015), *calcium-dependent protein kinase* (*CDPK*), *lycopene ε-cyclase* (*IBLCY-ε*) ortholog, *GPD*, trehalose synthesis protein, and regulatory proteins such as *StEREBP*, *CBF*, and *StRD22* (Byun et al., 2007), *MeALDH*, *MeZFP*, *MeMSD*, *MeRD28* (Muiruri et al., 2021), *mitogen-activated protein kinase kinase kinases* (*MeMAPKKKs*), and *KUP* (*MeKUP*) family genes (Ye et al., 2017; Ou et al., 2018), *respiratory burst oxidase homolog* (Rboh) (Li et al., 2015), *WRKY53* (Wang et al., 2017), *slow osmotic stress 5* (Gao et al., 2011), *late embryogenesis abundant* (*IbLEA14*) (Park et al., 2011), iron–sulfur cluster scaffold protein gene *IbNFU1*, a/b-hydrolase gene *IbMas* (Liu et al., 2014a; Liu et al., 2014b), myo-inositol-1-phosphate synthase gene *IbMIPS1* (Zhai et al., 2016), salt-induced methyltransferase gene *IbSIMT1* (Liu et al., 2015), and stress-inducible promoters (Ryu et al., 2009) can be potential candidates for editing to achieve this objective.

The following genes are found to be involved in osmotic regulation, stomatal dynamics, and ABA/auxin/ethylene signaling like *AtHDG11* (Ruan et al., 2012), *AtOST2* (Osakabe et al., 2016), *ARGOS8* in maize (Shi et al., 2017), *SlNPR1* (Li et al., 2019), and *OsERA1* (Tripathy et al., 2021). Orthologs of these genes in RTC species can be useful for developing drought tolerance in sensitive varieties. Being sensitive to extreme cold, cassava cultivation is confined to the tropics. Enhancement of cold tolerance in cassava is important for broadening its geographical distribution and to avoid yield loss that may occur due to unprecedented cold weather, in regions experiencing extreme cold during winter (like in North India between November and February) and at high-altitude locations. Certain cold-responsive genes and transcription factors described by Van Berkel et al. (1994), Kim et al. (2011), An et al. (2012, 2016), Cheng et al. (2019), Fan et al. (2012), Fan et al. (2015b), Park et al. (2015), and Li et al. (2018) can be validated in RTCs and appropriately manipulated for cold stress tolerance.

Appropriate manipulation of genes regulating the metabolic processes underlying water use efficiency, photosynthetic efficiency, transpiration rate, and epidermal conductivity may be effective for reducing yield loss due to drought stress, especially under current circumstances of erratic precipitation patterns (Hatfield and Dold, 2019). Genetic regulation of expression of root system architecture determinants, primarily those of lateral root, can be studied with the help of CRISPR/Cas9-induced mutations, and specific modifications in the respective genes would ensure nutrient availability even under stress conditions. This, in turn, will help maintain an optimum cellular concentration of certain mineral nutrients that is essential to facilitate key events of molecular mechanisms that play a significant role in alleviating the detrimental effects of drought stress like antioxidant defense and osmoregulation (Waraich et al., 2011).

### 4.8 Storability and post-harvest physiological deterioration of tubers

Enzymatic browning of potato and yam tubers induced by polyphenol oxidases (PPOs) during harvest and post-harvest procedures like transport, storage, distribution, and blanching is an important issue as it adversely affects the texture, flavor, and color of tubers, thereby reducing their marketability (Jia et al., 2015). PPOs cause rapid polymerization of phenols to form O-quinone which forms black precipitates in tubers. Previous attempts to prevent browning were RNAi based, most of which caused the knockdown of multiple *PPO*s and thereby impaired other cellular functions for which PPOs are required. The efficiency of CRISPR/Cas9-induced knockout of the *PPO* to yield non-browning mushrooms and apples is evidenced in different studies (Halterman et al., 2016; Nishitani et al., 2016; Waltz, 2016; Waltz, 2018). A similar strategy was adopted by González et al. (2020) that successfully prevented the enzymatic browning of potato tubers. In this study, the *StPPO2* gene which is responsible for most of the polyphenol oxidation of potato tubers was selected as the target. The CRISPR/Cas9 knockout mutants exhibited reduced *StPPO* activity and a decrease in enzymatic browning up to 69% and 73%. RNP-mediated transformation strategy produced transgene-free and non-browning potato. Another significant finding is that the editing of a single gene *StPPO2* from the whole *PPO* gene family is enough to prevent browning while the important functions of the rest of the *PPO* family genes remain unaffected.

Storability of tubers is affected by mechanical damage like bruising, crushing, and rupture during harvest and by physiological factors such as respiration, transpiration, dormancy, and sprouting (FAO, 1998). Chilling causes internal tissue breakdown, increased water loss, and susceptibility to decay and adversely affects culinary qualities and taste. Increased respiration at higher temperatures leaves tissues with insufficient oxygen and builds up toxic levels of carbon dioxide. This in turn results in cell death as in the case of black hearts formed in potatoes at high temperatures (<30°C) (FAO, 1998). Similarly, transpirational water loss from tubers affects product quality and marketability and causes economic loss. In addition to weight loss, shriveled skin texture due to water loss results in big peeling loss, affects the culinary quality, and makes produce less appealing to consumers. Modification of temperature-responsive genes and temperature-sensitive regulators of respiration and transpiration using genome editing techniques may alleviate these issues.

Sprouting causes water and dry matter loss from sweet potato, potato, and yam tubers. This renders tubers susceptible to pathogen attack and makes them unsuitable for further storage and marketing (FAO, 1998; Afek and Kays, 2004; Afek and Kays, 2010; Sugri et al., 2019). An endogenous phenolic growth inhibitor seen in yams, namely, batatasins, which play an important role in inducing and maintaining dormancy, seems to be a candidate target in this context (Passam and Noon, 1977). This will reduce the expenses for chemical sprout suppressants and specific low-temperature storage facilities. Cold storage period, as well as processing traits of potato, has been improved by TALEN-mediated silencing of vacuolar invertase (*VI*nv) gene which minimizes the formation of anti-nutrients during the processing of tubers by lowering the accumulation of reducing sugars (Clasen et al., 2016). CRISPR/Cas9 can be deployed for characterization and precise manipulation of similar candidates in RTCs for preserving the nutritional quality of the produce under cold storage conditions. Promotion of the curing process, suberization, cork cambial activity, activation of components of oxidative stress management like ROS scavenging enzymes, and modification of ABA and ethylene signaling will reduce post-harvest physiological deterioration, minimize respiration, reduce weight loss of tubers, prevent pathogen invasion, and reduce damage during storage (Imaseki et al., 1968; Cottle and Kolattukudy, 1982; FAO, 1998; Beeching et al., 2000). The following genes could be suitable editing targets in this regard: phenylalanine ammonia-lyase (PAL) enzymes, hydroxyproline-rich glycoproteins (HPRGs) (Reilly et al., 2007), tyramine hydroxycinnamoyl transferase (THT) (Negrel et al., 1993), ROS scavenging/oxidation-related enzymes, ascorbate peroxidase (APX) (Uarrota et al., 2016), mitochondrial alternative oxidase gene (AOX1A), copper/zinc superoxide dismutase (*MeCu/ZnSOD*), and catalase (*MeCAT1*) (Zidenga et al., 2012). *Feruloyl 6′*-*hydroxylase* (*F6′H*) (Lim, 2019), a gene that drives the biosynthesis of scopoletin is another candidate target for knockout so that discoloration of tubers can be prevented.

### 4.9 Reprogramming developmental patterns and metabolism for improved traits

Important metabolic processes like photosynthesis, carbon assimilation, carbon allocation in roots and tubers, starch biosynthesis and catabolism, carbohydrate metabolism, and senescence along with their regulatory networks determine attributes such as sink strength, storage root dry matter, and starch content and composition. So, the manipulation of the respective regulatory factors, transcription factors, and biosynthetic/catabolic enzymes can produce favorable features in RTCs. Some of the putative editing targets for this purpose can be *NRP1* (Chen et al., 2021), *cell wall invertase* (*CWI*) and *vacuolar invertase* (*VI*) (Jin et al., 2009), *ADP-glucose pyrophosphorylase* (*AGPase*), *AATP protein-encoding gene* (*IbAATP*) (Wang J. et al., 2016), *sucrose non-fermenting-1-related protein kinase-1* (*IbSnRK1*) (Jiang et al., 2013), *IbSRF1* (Tanaka et al., 2009), alpha- and beta-amylases (Kato and Uritani, 1976), and *IPT* (Zhang et al., 2010). Modification of growth pattern regulating genes, transcription factors, and hormonal and non-cell-autonomous signals through targeted mutagenesis provide an effective strategy to obtain desirable plant architecture like bushy plants in yams and short stems in sweet potato, which would ease management practices.

### 4.10 Improvement of root system architecture

The root system architecture (RSA) has a profound impact on the development and yield of storage roots and tubers and is determined by complex and interconnected regulatory networks (Khan et al., 2016). Lateral roots (LRs) are an important component of the RSA, and their growth, development, and branching pattern influence the water absorption capacity, nutrient use efficiency, and storage tuber development in certain RTCs (Villordon et al., 2012; Khan et al., 2016). Homeostasis and interplay of auxin, cytokinin, and ABA, certain transcription factors (e.g., KNOX) (Overvoorde et al., 2010; Goh et al., 2012), primary cambial activity, rapid division and proliferation of metaxylem cells and vascular cambium cells, lignin metabolism, and stele lignification (Kim et al., 2002; Noh et al., 2010; Noh et al., 2013; Khan et al., 2016) also have an impact on the RSA. Factors regulating these processes could be manipulated for desirable modifications. The following genes can be considered putative targets for editing: *SRF* family genes (e.g., *SRF6*) (Tanaka et al., 2005), *knotted-like homeobox* (*KNOX*) family genes (e.g., *POTH1* in potato), *Ibkn1*, *Ibkn2*, and *Ibkn3* (Tanaka et al., 2008), *MeKNOX5* and *MeKNOX8* (Guo et al., 2014), MADS-box family transcription factors [e.g., *IbMADS1* (Ku et al., 2008) and *POTM1* (Rosin et al., 2003)], which synchronize storage root development predominantly by their phase-specific differential expression to regulate signaling of phytohormones, expansins (*IbEXP1*) (Noh et al., 2013), GA-related biosynthesis genes (*KS* and *GGPS1*), polar auxin transport genes (*AUX1* and *PIN1*), cytokinin responsive factor (*CRF1*), ethylene signaling genes (*ERF/CEJ1_3227*) (Sojikul et al., 2015), GA regulating *StBEL5* (Chen et al., 2004), and *FLOWERING LOCUS T* (*FT*) (Abelenda et al., 2014). The possibility of obtaining the preferred shape and form of tubers amenable for harvesting and processing by modification of genes determining the patterns of storage root development may also be investigated.

### 4.11 Biofuel-oriented genomic modifications

The development of traits favorable to biofuel production will help enhance RTCs' contribution to supplement the depletion of fossil fuels and combat environmental pollution and global warming. Genome editing can be applied to develop cassava and sweet potato varieties with improved traits like high fermentable sugar content and modified processing characteristics like amenability to hydrolysis and fermentation. This renders them fit for biofuel production by facilitating their efficient conversion to biofuels (Thatoi et al., 2016). Precise manipulation of carbon partitioning into starch storage organs, the source–sink relationship, the starch biosynthesis pathway, and ATP availability for starch synthesis would be useful for boosting starch synthesis, thereby providing more feedstock for biofuel production. Engineering lignin metabolism to reduce lignification of storage organs through manipulation of lignin monomer synthesis or appropriate regulation of lignin degradation pathways may be useful (Weng et al., 2008). Optimal regulation of other sugar metabolizing pathways to channel photoassimilated carbon into starch synthesis can also be effective for this purpose (Smith, 2008). While multiple cross-talking metabolic pathways are manipulated for obtaining tubers rich in starch, targeted mutagenesis should be optimally engineered without hampering the normal growth and development of plants.

Apart from extensively cultivated and consumed cassava, sweet potato, and potato, genome editing applications have expanded to yam species and carrot along with the advancements in deciphering sequence data and development of genetic transformation and regeneration protocols. Syombua et al. (2021) established CRISPR/Cas9-based gene editing in a West African preferred cultivar, *D. rotundata*, by targeting *phytoene desaturase*, the typical target gene that functions as a visual marker of gene editing. An editing efficiency of 83.3% by expressing two gRNAs under a U6 promoter derived from another *Dioscorea* species, *Dioscorea alata*, through *Agrobacterium*-mediated transformation was obtained (Nyaboga et al., 2014). The successful establishment of an efficient CRISPR/Cas9 editing system with a specific promoter provides opportunities for improving traits in yam species.

Similarly, Klimek-Chodacka et al. (2018) reported the first successful gene editing in carrot. CRISPR/Cas9-mediated knockout of *flavanone-3-hydroxylase* (*F3H*) gene, a critical component of the anthocyanin biosynthesis pathway caused discoloration of carrot calli. Despite the identification of AteCas9 as the most efficient effector for inducing mutations, they could not regenerate edited plants. In another study by Xu et al. (2019), two genes performing critical functions in chlorophyll and anthocyanin biosynthesis, namely, *DcPDS* and *DcMYB113*-like, the genes present in orange and purple carrot, respectively, were knocked out by targeted editing. Albino and purple depigmented plantlets were regenerated with an editing efficiency of 35.3% and 36.4%, respectively, by editing *DcPDS* and *DCMYB113*-like gene. This study also identified that AtU6-29 can be an efficient promoter for sgRNA expression in carrot.

The application of CRISPR/Cas-mediated gene editing in RTCs for various purposes, as described so far, is summarized in Table 1.

**TABLE 1:** List of root and tuber crops modified with the CRISPR/Cas system.

	Plant species	Trait	Target gene(s)	Transformation system	CRISPR/Cas system	Reference
1	Cassava	Proof of concept (albino phenotype)	*Phytoene desaturase (PDS)*	*Agrobacterium*—FEC transformation	CRISPR/Cas9	Odipio et al. (2017)
2	Potato	Proof of concept	*StIAA2*	*Agrobacterium*—stem segment transformation	CRISPR/Cas9	Wang et al. (2015)
3	*Dioscorea rotundata*	Albino phenotype—proof of concept	*Phytoene desaturase* (*PDS*)	*Agrobacterium*—nodal explant transformation	CRISPR/Cas9	Syombua et al. (2021)
4	Cassava	Acyanogenic cassava	*CYP79D1* and *CYP79D2*	*Agrobacterium*—FEC transformation	CRISPR/Cas9	Gomez et al. (2023)
5	Cassava	Acyanogenic cassava	*CYP79D1*	*Agrobacterium*—FEC transformation	CRISPR/Cas9	Juma et al. (2022)
6	Potato	Elimination of steroidal glycoalkaloids—α-solanine and α-chaconine	*St16DOX*	*Agrobacterium*—hairy root transformation	CRISPR/Cas9	Nakayasu et al. (2018)
7	Potato	Abiotic stress tolerance (water stress)	*CYCLING DOF FACTOR 1* (*StCDF1*) and *StFLORE*	*Agrobacterium*-mediated transformation	CRISPR/Cas9	Gonzáles et al. (2020)
8	Potato	Cyanide respiratory pathway inhibition and abiotic stress tolerance	*StAOX1* (*Alternative oxidaseI*)	*Agrobacterium*-mediated transformation	CRISPR/Cas9	Hua et al. (2020)
9	Potato	*Potato virus Y*. resistance	*P3*, *CI*, *NIb*, or *CP* of PVY	*Agrobacterium* transformation of protoplast	CRISPR/Cas9	Zhan et al. (2019)
10	Cassava	*Ugandan cassava brown streak virus* (UCBSV)	*Novel cap-binding protein1* (*nCBP-1*) and *nCBP-2*	*Agrobacterium*—FEC transformation	CRISPR/Cas9	Gomez et al. (2019)
11	Sweet potato	Sweet potato virus disease (SPVD) resistance	*RNase3* of *sweet potato chlorotic stunt virus* (SPCSV)	*Agrobacterium* transformation	CRISPR/Cas13d	Yu et al. (2022)
12	Potato	Resistance to PLRV, PVY, PVS, and PVX	*CP* gene of PLRV, PVY, PVS, and PVX	*Agrobacterium* transformation	CRISPR/Cas13d	Zhan et al. (2023)
13	Cassava	ACMV resistance	*AC2* and *AC3* of ACMV	*Agrobacterium* transformation	CRISPR/Cas9	Mehta et al. (2019)
14	Potato	PVY resistance and salt and osmotic stress tolerance	Coilin gene	Particle bombardment	CRISPR/Cas9	Makhotenko et al. (2019)
15	Cassava	Bacterial blight resistance	*MeSWEET10a* and EBE_TALE20_	*Agrobacterium*—FEC transformation	CRISPR/Cas9	Wang et al. (2022)
16	Cassava	Bacterial blight resistance	*MeSWEET10a* and TAL20 EBE	*Agrobacterium*—FEC transformation	CRISPR/Cas9	Elliott et al. (2023)
17	Potato	Late blight resistance	*MLO1*, *HDS* homolog, *AtTTM2* homolog, *StDND1*, *StCHL1*, *StDMR6-1*, *StDMR6-2*	*Agrobacterium*—leaf explant transformation	CRISPR/Cas9	Kieu, et al. (2021)
18	Cassava	Glyphosate resistance	*5-enolpyruvylshikimate-3-phosphate synthase* (*EPSPS*)	*Agrobacterium*—FEC transformation	CRISPR/Cas9	Hummel et al. (2018)
19	Potato	Imidizolinone resistance	*Acetolactate synthase* (*ALS1*)	*Agrobacterium*—leaf explant transformation	CRISPR/Cas9	Butler et al. (2015), Butler et al. (2016)
20	Potato	Amylose-free starch	*Granule-bound starch synthases* (*GBSSI*)	RNP-mediated protoplast transformation	CRISPR/Cas9	Andersson et al. (2018)
21	Potato	Amylose-free starch	*Granule-bound starch synthases* (*GBSSI*)	*Agrobacterium* and protoplast transformation	Cytidine base editor	Veillet et al. (2019)
22	Cassava	Low amylose starch	*Protein targeting to starch* (*MePTST*) and *GBSSI*	*Agrobacterium*—FEC transformation	CRISPR/Cas9	Bull et al. (2018)
23	Sweet potato	Starch granule morphology	*IbGBSSI* and *IbSBEII*	*Agrobacterium*—embryogenic calli transformation	CRISPR/Cas9	Wang et al. (2019)
24	Potato	Non-browning tubers	*Polyphenol oxidase2* (*PPO2*)	RNP-mediated protoplast transformation	CRISPR/Cas9	González et al. (2020)
25	Potato	Self-compatibility in diploid cultivar	*S-RNase*	*Agrobacterium*—leaf explant transformation	CRISPR/Cas9	Enciso-Rodriguez et al. (2019)
26	Cassava	Accelerated flowering	*FLOWERING LOCUS T1* (*FT1*)	*Agrobacterium*—FEC transformation	CRISPR/Cas9	Odipio et al. (2020)
27	*Dioscorea zingiberensis*	Proof of concept (squalene content)	*Farnesyl pyrophosphate synthase* (*FPS*)	*Agrobacterium*-mediated transformation	CRISPR/Cas9	Feng et al. (2018)
28	Carrot	Discoloration of calli	*F3H*	*Agrobacterium*—callus transformation	CRISPR/Cas9	Klimek-Chodacka et al. (2018)
29	Carrot	Albino and depigmented plants	*DcPDS* and *DcMYB113*	*Agrobacterium*—seedling explant transformation	CRISPR/Cas9	Xu et al. (2019)
30	Potato	Proof of concept	*StALS1* and *StALS2*	*Agrobacterium* transformation	Prime editing	Veillet et al. (2020)
31	Potato	Resistance to late blight disease	*StPM1*		CRISPR/Cas9	Bi et al. (2023)

**FIGURE 2:**
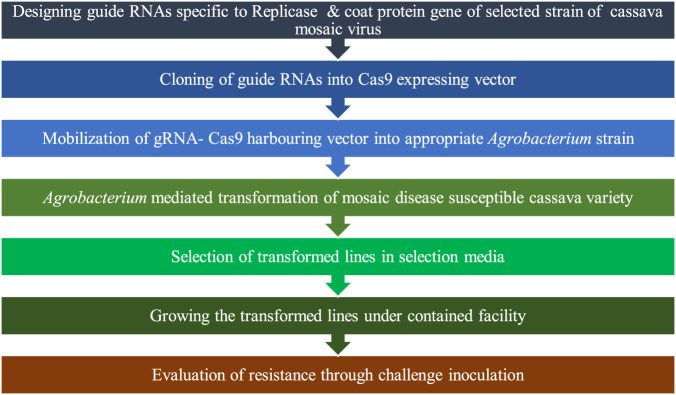
Developing cassava mosaic disease resistance through gene editing.

**FIGURE 3:**
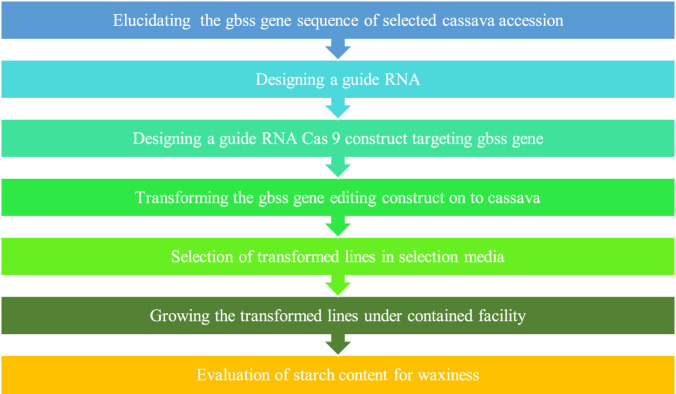
Waxy starch from cassava through gene editing.

The Central Tuber Crops Research Institute, Thiruvananthapuram, India, is involved in research activities for the improvement of agronomic traits and resilience, for value addition, and in devising efficient management practices in tropical RTCs. The emerging advanced techniques have been deployed to fulfill the research objectives concerning RTCs. Currently, attempts to apply CRISPR/Cas9-mediated gene editing in two aspects of RTC improvement is ongoing, i.e., starch modification and disease resistance. Research in making modified starch from South Indian farmer-preferred landraces and cassava mosaic virus-resistant cultivars is ongoing, and the strategy adopted for this is given in Figures 2, 3.

### 4.12 Transformation and regeneration

An efficient system for the delivery and expression of CRISPR/Cas9 components to host plants and regeneration of edited lines is a primary requisite for genome editing in any species. This is accomplished either by stable integration or transient expression of Cas9 and gRNA constructs in the host cells. The feasibility of host plant transformation with target-specific CRISPR/Cas9 constructs is of paramount importance for the successful application of this genome editing technique for the improvement of RTC species in the foregoing aspects. The lack of efficient transformation and regeneration protocols is a major limiting factor in utilizing the potential of gene editing in various RTCs. Although cassava and sweet potato are comparatively improved regarding the availability of protocols, their recalcitrant nature renders the process difficult in many cases. The efficiency of transformation and regeneration is species and genotype-dependent (Borrelli et al., 2018).

*Agrobacterium*-mediated transformation, PEG-induced or electroporation-mediated protoplast transfection, biolistics (Shan et al., 2013; Jacobs et al., 2015; Svitashev et al., 2015), and virus-based delivery system (Butler et al., 2016; Gil-Humanes et al., 2017) are the methods for the delivery of CRISPR/Cas9 expression cassette into the host plant cell. Embryogenic calli are considered ideal for *Agrobacterium*-mediated transformation of cassava (Bull et al., 2009; Jayaseelan et al., 2017), sweet potato (Yu et al., 2007), and elephant foot yam (Kamala and Makeshkumar, 2014) and axillary bud explants are efficient for *Dioscorea* spp. (Nyaboga et al., 2014). Syombua et al. (2022) have found that friable embryogenic calli also can be developed as suitable target explants for the genetic transformation of *Dioscorea* spp.

Direct introduction of pre-formed functional sgRNA-Cas9 ribonucleoprotein complex (RNP) into host cells enables non-transgenic delivery (Woo et al., 2015; Svitashev et al., 2016; Liang et al., 2017). This strategy is useful only for species that are amenable to protoplast regeneration. However, there are chances of somaclonal variations in protoplast-regenerated plants. Biolistic or protoplast transformation, either with RNP or RNA intermediates, i.e., Cas9 coding sequence and DNA sequence corresponding to sgRNA, mostly favor HDR-mediated repair over NHEJ, thereby providing gene insertion options (Borrelli et al., 2018). Transiently expressed CRISPR/Cas9 DNA or transiently expressed CRISPR/Cas9 in *in vitro* transcripts can also be delivered to immature embryos of RTC species for which protoplast transformation is less efficient or shows recalcitrance to protoplast regeneration (Zhang et al., 2016). Potato is exceptional in this regard with its efficient protoplast regeneration potential. It is evident from the previously discussed cases of CRISPR/Cas9-mediated editing of the protoplast and recovery of non-transgenic-edited potato plants (Andersson et al., 2018; Veillet et al., 2019; González et al., 2020).

Viral vectors are known for their utility in virus-mediated overexpression of proteins and virus-induced gene silencing for transient knockdown of target genes. Recent studies have demonstrated the potential of virus vectors in mediating the delivery of gene editing components. Virus-induced gene editing (VIGE) is manifested in two ways, either by delivering guide RNA into a Cas overexpressing plant or by simultaneous delivery of both guide RNA and Cas9 in a single vector or two distinct vectors (Yin et al., 2015). Viral vectors are advantageous for the rapid expression of the CRISPR toolbox at the whole plant level with a simple inoculation procedure, eliminating the requirement for a complex and time-consuming transformation process and providing a transgene-free editing platform. Starting from *Tobacco rattle virus* (TRV), several viruses like *Tobacco mosaic virus* (TMV), *Beet necrotic yellow vein virus* (BNYVV), *Barley stripe mosaic virus* (BSMV), *Foxtail mosaic virus* (FoMV), *Potato virus X* (PVX), *Cabbage leaf curl virus* (CaLCuV), *Pea early browning virus*, and DNA replicons derived from deconstructed geminivirus (GRV) have been successfully deployed as CRISPR/Cas vectors, mostly in model plants (Baltes et al., 2014; Cermak et al., 2015; Cody et al., 2017; Ali et al., 2018; Hu et al., 2019; Jiang et al., 2019; Mei et al., 2019; Ellison et al., 2020). TMV is an excellent vector system providing versatile editing options like multiplex editing and heterologous gene expression (Cody et al., 2017). Ariga et al. (2020) devised a DNA virus vector system enabling efficient virus-free and transgene-free editing using PVX. PVX vector successfully induced mutations in two target genes in *Nicotiana benthamiana* through agroinfiltration. Edited lines free of transgene and viral DNA were regenerated from agroinfiltrated leaves. The amenability of this PVX vector system for base editing was also demonstrated using a nickase SpCas9 fused with cytidine deaminase (nSpCas9-NGv1-AID).

Recently, the *cassava common mosaic virus* (CsCMV) has been identified as a potential VIGE vector. CsCMV is a *Potexvirus* with single-stranded RNA genome. Characteristics like mild infection symptoms and rapid Nimble Cloning (NC) of target fragments into the viral genome render it suitable for VIGE. Tuo et al. (2023) employed CsCMV vector, pCsCMV2-NC to deliver two guide RNAs (gMePDS_1_ and gMePDS_2_) targeting exon 13 of *MePDS* into Cas9 overexpressing cassava. This produced albino phenotypes having indel mutations in the target regions of gMePDS_1_ and gMePDS_2_ with an editing efficiency ranging from 17.3% to 23.9% and 45.7%–47.4%, respectively. This strategy is an efficient alternative to time-consuming and laborious transformation-based gene editing in cassava. Ma et al. (2020) demonstrated the successful deployment of *Sonchus yellow net rhabdovirus* (SYNV), a *Rhabdovirus* as a CRISPR/Cas9 delivery vector. In tetraploid tobacco, chromosome deletions and multiplex editing with SYNV vector generated mutations with 57% efficiency. Thus, SYNVs can be promising vectors for CRISPR/Cas9 editing of RTC species, particularly because of their ability to carry large gene fragments.

Although viral vectors enable efficient delivery of gRNA and Cas, further optimization of gRNA expression and a suitable promoter is required to address the low efficiency of mutations. The large size of Cas9 is a challenge in VIGE due to the limited cargo capacity of viruses and the instability of viral vectors carrying long gene inserts (Avesani et al., 2007). Since the viral vectors described are non-seed transmissive, the inheritance of mutations induced by them is an important bottleneck. *In vitro* regeneration of plants from virus-infected, edited plant tissue is one way to produce transgene-free edited plants with heritable mutations. Another strategy involves the delivery of guide RNA fused with a mobile *FLOWERING LOCUS T* mRNA into germline cells. The efficiency of this strategy has been proved in model plants such as *A. thaliana*, *N. benthamiana*, and *N. attenuate* with PVX, *Tobacco rattle virus*, and *Cotton leaf crumple virus* vectors (Ellison et al., 2020; Lei et al., 2021; Uranga et al., 2021). Germ cell localization and editing activity of gRNA through its coupling with germline homing *FT* mRNA have to be evaluated in cassava and other RTC species.

Direct delivery of DNA-free RNP by evolving techniques like site-specific nuclease-binding cell-penetrating peptides (CPPs) for plant systems has to be assessed (Rádis-Baptista et al., 2017; Metje-Sprink et al., 2019) for its application in RTCs. The delivery method should be optimized for RTCs by considering several factors such as species, the purpose of editing, i.e., whether mutagenesis or a repair matrix with a template DNA is aimed, and the developmental stage at which transformation is to be performed (Borrelli et al., 2018).

Regeneration of fertile plants from cells or tissues transformed by the CRISPR/Cas9 tool is of prime importance for field-level application of the technology. Genotype dependence, time consumption, and the lack of efficient protocols for the regeneration of mature plants through cell/tissue culture used to be the major limitations for the application of biotechnological strategies for the improvement of most of the RTC species. This renders the genetic engineering of RTCs underdeveloped.

The development of a standardized transformation and regeneration protocol for cassava took several years. Attempts were initiated in the 1980s when a group of scientists succeeded in generating the first transgenic cassava calli (Calderon-Urrea, 1988). The most important point in cassava transformation is the production of friable embryogenic callus (FEC), the target tissue. This requires a series of steps like the *in vitro* culture of plants, induction of a primary somatic embryo and then a secondary cyclic somatic embryo and FEC, followed by repeated subculture to isolate and purify the healthy FEC. This process is time-consuming (∼4 months), and the success depends on the genotype and expertise. Repeated subcultures affect the quality and viability of the FEC and cause an imbalance of endogenous hormone levels. This affects the regeneration potential and morphology of the regenerated plants. In addition, the chances of somaclonal variation of FCEs are high due to repeated subcultures with constant exposure to auxin (Chavarriaga-Aguirre et al., 2016). The availability of high-quality FEC is essential for the regeneration of a sufficient number of transgenic lines. However, after the first published reports of cassava FEC transformation, there were several attempts to improve the transformation system together with standardization of a very tedious, yet inevitable part of cassava regeneration, i.e., the induction and maintenance of FEC (Schöpke et al., 1993; Sarria et al., 1995; Taylor et al., 1996; Bull et al., 2009; Liu et al., 2011). This led to the application of transgenic technology for improving agronomic traits in cassava. Initially, cassava transgenic research remained confined to model cultivar 60444. It was extended to cultivated cassava landraces following the optimization of key factors determining the success of FEC generation, transformation, and regeneration (Fregene and Puonti-Kaerlas, 2001; Taylor et al., 2002; Bull et al., 2009; Taylor et al., 2012). Robust FEC transformation protocols were developed for African farmer- and industry-preferred landraces like TME3, TME7, TME14, T200, TME204, Ebwanatereka, Kibandameno, Serere, and AD001 (Vanderschuren et al., 2012; Zainuddin et al., 2012; Chetty et al., 2013; Nyaboga et al., 2013; Nyaboga et al., 2015; Chauhan et al., 2015; Elegba et al., 2021). Recently, Wang et al. (2022) reported an efficient transformation and regeneration system for South Chinese cassava cultivar 8 (SC8). With the optimization of *Agrobacterium* cell density, co-cultivation duration, optical density of *Agrobacterium* culture, acetosyringone concentration, and humidity of FEC specific to the cultivar, a high transformation and regeneration efficiency was observed. CRISPR/Cas9-mediated editing of *MePDS* gene was also achieved with this protocol (Wang et al., 2022). Even though all these seem to represent significant progress achieved in cassava genetic transformation and regeneration, successful standardizations have been established for only a fraction of the total cassava landraces.

Zhang et al. (2000) have demonstrated the successful transformation of cassava somatic cotyledons by particle bombardment. Protocols for PEG-mediated protoplast transfection with target-specific RNP and subsequent regeneration of mature plants from mutated single cells via tissue culture techniques have been optimized for some crops. Standardized protocols for cassava protoplast transformation could not provide plant regeneration (Wu et al., 2017). Integration of the established protocols by Sofiari et al. (1998) and Shahin and Shepard (1980) for cassava protoplast regeneration with a transformation system may efficiently resolve this constraint. However, the regeneration of transgenic plants from isolated protoplasts transformed by PEG or particle bombardment is difficult. *Agrobacterium*-mediated transformation is preferred to obtain efficient transformation and better regeneration frequencies in such cases.

Studies have shown that transformation efficiency can be improved by modifying conditions of tissue culture and *Agrobacterium* infection. This includes modification of media composition like the addition of amino acids or altering plant growth regulators, changing the optical density of *Agrobacterium* culture, the addition of cephalosporin before *Agrobacterium* inoculation, modified co-cultivation duration, and acetosyringone concentration (Chavarriaga-Aguirre et al., 2016). Brand et al. (2019) investigated the role of *Arabidopsis LEAFY COTYLEDON 1* (*LEC1*) and *LEC2* orthologs in cassava model cultivar 60444. The result of the study is consistent with the well-established significant regulatory role of LEC in embryogenesis. A detailed analysis of the differentially expressed genes and metabolic profiles, and cytological and epigenetic modifications, in FECs have provided insights into regulatory networks operated in SE to FEC conversion and FEC development (Ma et al., 2015). The study also indicates epigenetic and genetic expression patterns influencing somaclonal variations in FECs. Information from such studies is useful to optimize the transformation and regeneration protocols in cassava. However, the lack of sufficient information about cassava embryogenesis marker genes, embryogenesis-specific regulatory genes, and genetic and epigenetic factors influencing dedifferentiation and totipotency induction in somatic cells of cassava curbs the attempts to fill the research gap in this regard. The genetic and molecular basis of heavy dependence on genotype, explant type, and culture conditions for cassava transformation and regeneration has not been completely elucidated yet. The lack of these details is a major limitation in addressing the complexities of cassava genetic engineering.

Initial attempts for sweet potato transformation employed particle bombardment and electroporation and had low regeneration frequency of transformed embryogenic calli or transient expression (Prakash and Varadarajan, 1992; Newell et al., 1995; Gama et al., 1996; Song et al., 2004). Further trials of hairy root transformation using *A. rhizogenes* and *A. tumefaciens* showed low transformation frequency (Otani et al., 1998). Later, efficient protocols for *A. tumefaciens*-mediated transformation system for sweet potato were developed by various researchers (Song et al., 2004; González et al., 2008; Yang et al., 2011). Most of them used leaf explants, whereas some used stem and axillary bud explants. Embryogenic suspension culture was shown to be suitable for transformation with *A. tumefaciens* strains A208SE and LBA4404 (Zhai and Liu, 2003; Jiang et al., 2004) and plant regeneration. Yu et al. (2007) optimized the protocol for sweet potato transformation with *A. tumefaciens* strain EHA105 for sweet potato cv. *Lizixiang* and had successfully applied it in several other genotypes (Fan et al., 2012; Wang Y. N. et al., 2016). The regenerative potential of sweet potato is high compared to cassava and is not FEC dependent. However, sweet potato transformation and regeneration is also genotype-dependent and hence require specifically optimized protocols in highly recalcitrant cultivars. Like cassava, genetic and molecular regulatory factors determining regeneration and transformation have to be investigated and appropriately manipulated to augment the gene editing-based crop improvement programs in sweet potato.

The recently developed cut–dip–budding (CDB) delivery system (Cao et al., 2023) is a breakthrough in sweet potato transformation. It does not require tissue culture and regeneration and can surpass all the bottlenecks of previous protocols. In this method, the apical shoot of sweet potato is inoculated with *A. rhizogenes*, then planted in vermiculite, and kept in the incubator. Once the stem starts developing roots, they can be screened for transgene expression and transferred to the soil. In the soil, roots are enlarged and some form tuberous roots as well. Both roots and tuberous roots are cut into pieces and planted in the soil. All transgenic roots efficiently produce transgenic shoots. The amenability of this transformation system for CRISPR/Cas9 editing has also been evaluated. Interestingly, the CDB system is genotype-independent and can have promising impacts in the future. The amenability of the CDB system in other RTCs has to be evaluated. Being vegetatively propagated in nature, CDB transformation can be highly valuable for RTCs.

Despite their significant nutritional potential, yam species remains comparatively unnoticed among other tuber crops like cassava and sweet potato. Hence, the genetic engineering of these orphan crops has lagged compared to major root crops to a large extent. There were no reports of genetic transformation and regeneration of yam species until 2014. The regeneration system for two species: *D. rotundata* and *D. alata* was first developed by Adeniyi et al. (2008). Anike et al. (2012) standardized the regeneration protocol for *D. rotundata*, *D. cayenensis*, and *D. alata* from petiole explants, and Manoharan et al. (2016) demonstrated the regeneration of *D. rotundata* from axillary bud explants. The first-ever transient transformation of *D. alata* was done by Tor et al. (1993) by particle bombardment of cell suspension culture. Protocols have been developed for PEG-mediated protoplast transformation of *D. alata* and *Agrobacterium* transformation of *D. rotundata* (Tor et al., 1998; Quain et al., 2011). However, none of these could come up with successful regeneration of transgenic plants. The first successful *Agrobacterium*-mediated genetic transformation and plant regeneration of a *Dioscorea* species was established in a medicinal species *D. zingiberensis* (Shi et al., 2012). Nyaboga et al. (2014) developed *Agrobacterium* transformation and regeneration system for *D. rotundata*. This efficient and reproducible transformation and regeneration protocol uses axillary bud explants and requires a duration of approximately 4 months to get transgenic plants. Recently, a genetic transformation protocol for another medicinally important yam species, *D. opposita*, known as Chinese yam, was also developed (Li R. et al., 2019). Fukino (2000) and He et al. (2004, 2010) developed genetic transformation protocols for taro *C. esculenta* (L.), an important staple crop in Pacific islands. The former employed particle bombardment, whereas the latter used *Agrobacterium*-mediated transformation. However, these protocols are of very low efficiency and hence there is a need for robust ones. Similarly, elephant foot yam, another nutritionally important but less recognized RTC, lacks an efficient regeneration system although a transformation protocol has been optimized by Abraham et al. (2021).

*De novo* meristem induction system using developmental regulators is a suggested method for overcoming transformation and regeneration barriers in yams. This system involves the delivery of CRISPR reagents/constructs and developmental regulators like *WUSCHEL2* (*Wus2)* or *SHOOTMERISTEMLESS* (*Stm*) or *BABY BOOM* (*Bbm*) to the nodal explants (Maher et al., 2020). Developmental regulators will induce meristem formation from these nodal explants, followed by the growth of shoots with targeted edits. Although Ghogare et al. (2021) have developed protoplast editing protocols with high editing efficiency for yams, they could not achieve efficient plant regeneration. Transformation and regeneration systems for many beneficial yam species are not available yet. Continuous attempts to establish genetic transformation and regeneration systems for economically important RTC species that are neglected by mainstream research will enhance the possibilities of expanding their beneficial applications by employing gene editing.

After the transformation and regeneration, selection of transgene-free lines from the segregating progeny population is done, and this avoids the disadvantages like random integration of transgene, constitutive expression of unwanted transgenes, and off-target editing. The elimination of transgene by gametic segregation and subsequent recovery of Cas9 null-segregants is time-consuming and difficult in vegetatively propagated RTCs. Hence, the DNA-free RNP delivery system is particularly advantageous for RTCs to generate non-transgenic CRISPR/Cas9-edited lines. Recovered edited lines have to be subjected to molecular and phenotypic screening for the confirmation of on-target mutation, off-target effects, and desired trait integration/establishment.

### 4.13 Off-target effects of editing and strategies for improving specificity

Even though the CRISPR/Cas9 tool is regarded as highly specific, in some instances, off-target regions can also be recognized and cleaved by Cas9, resulting in unwanted damage to the genome that may or may not reflect in the phenotype. Although off-target events are not observed in most of the plant species edited with CRISPR/Cas9 (Nekrasov et al., 2013), the incidence of any such rare event can be overcome by crossing mutants with wild-type plants (Xie and Yang, 2013). Off-target effects can be either expected or unexpected. The former occurs with genome regions having high sequence similarity to the target, and the latter occurs in unrelated genome regions. Unexpected off-target cleavages usually do not occur at a frequency above the spontaneous mutation rate of plants (Borrelli et al., 2018). Strategies like optimal gRNA designing, target selection, and Cas9 expression (Fujii et al., 2013), use of truncated guide RNAs, high-fidelity Cas9 (Fu et al., 2014; Osakabe et al., 2016), modified endonuclease components like paired Cas9 nickases (Cas9D10A) or CRISPR RNA-guided Fok I nucleases (RFNs), and the addition of guanine residues to 5′ of gRNA (Fauser et al., 2014; Schiml et al., 2014; Tsai et al., 2014; Mikami et al., 2016) are recommended for reducing off-target effects. RNP-mediated delivery of CRISPR components is a better strategy for reducing off-targeting, particularly because of the non-integration of the CRISPR/Cas9 transgene, less functional time, and fast degradation of the RNP complex (Liang et al., 2017). Species-specific and purpose-specific optimization of target selection, gRNA design, choice of endonuclease, and transformation method are essential for obtaining efficient CRISPR/Cas editing in RTCs. Factors like binding strength of gRNA to target, chromatin structure, chromatin accessibility, and the epigenetic environment of target loci also have to be considered, as they have been found to influence the efficiency of target binding, Cas9 cleavage activity, and specificity of mutagenesis (Kuscu et al., 2014; Wu et al., 2014).

### 4.14 Transgene-free editing opportunity

CRISPR/Cas9-mediated genome editing has revolutionized plant genetic engineering by providing an efficient strategy for generating non-transgenic plants with improved traits of interest. This transgene-free editing is the highlighting feature that projects this tool as the most appropriate one for genome manipulation of crop plants. The mutations induced in genomic sequences by the CRISPR/Cas-mediated NHEJ repair mechanism is not different in any way from those resulting from the conventional crossing process, where DNA sequence variation is created by meiotic crossing over or the random mutagenesis induced by artificial mutagens or naturally occurring spontaneous mutations. Similarly, the HDR-mediated insertion of foreign genes into a genome is the same as the introgression of genes from a donor parent to offspring, which occurs during the artificial hybridization process. Since the regulations in many countries are product-focused and not process-based, crop plants edited with CRISPR/Cas9 are not included under genetically modified crops and are free of GM regulations. The regulations are applicable only if a gene encoding a toxin or any allergen is incorporated into a crop of interest (Duensing et al., 2018). CRISPR/Cas9-edited mushrooms, flax, and soybeans have obtained approval for marketing from US regulatory authorities and the FDA (Kim and Kim, 2016; Waltz, 2018).

### 4.15 Keeping pace with recent advancements: prime editing

Prime editing is a recent advancement in gene editing and is highlighted as powerful and efficient enough to circumvent the comparatively low efficiency of CRISPR/Cas9 in creating precise and predictable base substitutions. It can have promising implications in precision breeding by targeted induction of desired polymorphisms. Although base editors provide adenine/cytosine base conversions, there are certain drawbacks, like limited target site choice due to the PAM requirement, the occurrence of bystander mutations, and limited outcome diversity. Prime editing is a recently devised search and replace system in which a prime editing guide RNA (pegRNA) directs a prime editor to the target site. Prime editor is a fusion protein made of a *S. pyogenes* Cas9 nickase (SpnCas9 H840A) and an engineered *Moloney murine leukemia virus* reverse transcriptase (M-MLV RT). A separate sgRNA is deployed to introduce a nick in the non-edited strand so that the DNA repair is directed to that strand using the edited strand as the template. Once the M-MLV RT makes the first cut at 3 bp upstream to PAM on the non-target strand, the 3′-extension of pegRNA, comprising the primer binding site and reverse transcription sequence, facilitates the introduction of predefined modifications at the target site (Anzalone et al., 2019; Kantor et al., 2020). This is particularly advantageous when a single point mutation is enough to achieve the desired phenotype instead of the loss of function of the whole gene. However, prime editing is still in its emerging stage and requires further optimizations regarding its specificity. Plausible off-target modifications also have to be investigated.

The feasibility of this novel strategy has been demonstrated in potato as a proof of concept (Veillet et al., 2020). In this study, pegRNA was designed with spacers targeting *StALS1* and *StALS2*, a 13 bp primer binding site template, and a 15 bp RT site with 3 bp substitutions. Base substitutions are designated for the conversion of proline 186 to serine, and the modification of one of the three bases of the PAM site is to prevent further cleavage of the edited sequence by Cas9. Regenerated potato plants harbored mutations at the target site with 92% efficiency. Monoallelic substitutions were observed in the *StALS1* target site by employing a dicot codon-optimized prime editor. However, the efficiency of this prime editing is lower than previous base editing experiments in potato, where efficient tetra-allelic mutations were obtained (Veillet et al., 2019). This indicates the necessity of further optimization of prime editing systems for potato and other RTC species.

### 4.16 Underutilized RTCs and gene editing

Although RTCs are renowned as the world’s third most important crops, among them are many underutilized species. Unlike major RTCs like cassava, sweet potato, potato, yams, and aroids, these species are confined to their native areas and some are locally cultivated or grown in the wild in different parts of the world. They are nutritionally rich, and some possess medicinally and industrially valuable components. They provide food security to many tribal communities. Underutilized RTCs include Chinese potato (*S. rotundifolius*), arrowroot (*M. arundinacea*), Polynesian arrowroot (*Tacca leontopetaloides*), aerial yam (*Dioscorea bulbifera*), cocoyam (*C. esculenta*), yam bean, Queensland arrowroot, *Curcuma*, *Typhonium*, *Costus*, *Tacca*, and *Vigna* species (Archana et al., 2015). Chinese potato and arrowroot have attracted comparatively more attention, while others remain unrecognized. Chinese potato is consumed as a vegetable crop in South Indian states, and arrowroot starch is consumed as part of a healthy diet and has tremendous applications in the food industry. In addition to their high food-producing efficiency with high dry matter production per unit area per unit time, most of these underutilized RTCs are rich in minerals, vitamins, antioxidants, several beneficial secondary metabolites, and dietary fiber (Archana et al., 2015). Considering their high productivity, nutritional quality, and climate resilience, these underutilized RTCs are potential candidates for diet diversification and thereby provide significant support to mitigate malnutrition worldwide.

Despite their socioeconomic importance, most of the crop improvement-oriented research programs have ignored the underutilized RTCs. This may be due to a lack of knowledge and awareness about the nutritional benefits and industrial potential of these species. Underutilized RTCs require improvement of several attributes to increase their functionality, utility, and appeal. As in the case of Chinese potato, photosensitivity, low yield, and poor tuberization are major undesirable characteristics that have to be modified (Murugesan et al., 2020). Another important objective to be accomplished is the alleviation of anti-nutritional compounds and the enhancement of disease resistance. Phenol, alkaloid, oxalate, phytate, tannin, saponin, amylase inhibitors, and trypsin inhibitors present in certain *Dioscorea* species are anti-nutritional factors responsible for toxicity and bitterness. These anti-nutritional compounds make tubers acrid and cause symptoms like skin irritation and inflammation of the buccal cavity and throat after consumption (Padhan and Panda, 2020).

CRISPR/Cas9 can be employed to modify genes responsible for the synthesis of desired metabolites of industrial or nutritional value, anti-nutritional compounds, yield traits, and disease susceptibility. This would accelerate efforts for the inclusion of underutilized tuber crops in mainstream agriculture and dietary supplements. However, the major bottleneck in this aspect is the lack of an established genetic transformation and regeneration system. *In vitro* propagation methods have been developed for species like *S. rotundifolius* (Archana et al., 2015), *C. edulis* (Imai, 2008), *Typhonium trilobatum* (Das et al., 1999), and *Typhonium flagelliforme* (Sianipar and Maarisit, 2015), yet efficient callus induction and plant regeneration are reported only in *T. flagelliforme* and *S. rotundifolius*. Interestingly, the wild cultivar *Vigna* *vexillata* could regenerate plants from protoplasts isolated from seedling hypocotyl (Bhadra and Davey, 2005). Successful hairy root genetic transformation with *A. rhizogenes* is reported in *S. rotundifolius* and *V. vexillata*, but they failed to regenerate transgenic plants (Chandran and Potty, 2008).

The genetic engineering of underutilized tuber crops is an unfocused area of research. The lack of genomic sequence resources is an important constraint in this regard. In addition to this, there have not been sufficient attempts to evaluate the amenability of these crops to genetic engineering, develop efficient protocols for transformation and regeneration, and identify possible barriers in establishing the protocols. In this scenario, the application of gene editing for the improvement of underutilized RTCs is currently difficult. An extensive study is required to make underutilized RTCs amenable to genetic engineering approaches in the future. Elucidation of genome sequence and optimization of transformation–regeneration systems for these crops should be prioritized in future research. This is crucial to enable the application of advanced techniques for the improvement of underutilized RTCs.

## 5 Discussion

CRISPR/Cas9 offers a vast arena of specific and efficient gene editing options to accomplish the aforementioned modifications in RTCs in a time-saving and cost-effective manner. Most of the traits discussed previously as the target traits for improving cassava using CRISPR/Cas9-mediated genome editing is being addressed in the BioCassava Plus (BC+) program as well. BioCassava Plus initiative is envisaged to improve the health of Africans by employing modern biotechnology for biofortified cassava development and delivery in Sub-Saharan Africa (Sayre et al., 2011). The CRISPR/Cas9-based genome editing strategies can enhance the wide-ranging research programs dedicated for the improvement of RTC species that includes BC+. The characterization and development of modified CRISPR systems with Cas variants like Cpf1, FnCas9, Cas13a, or mutated SpCas9 like dCas9 or Cas9 nickase have expanded the range of applications for crop improvement owing to their characteristics like flexible target recognition features, simplified sgRNA organization, efficient multiplexing platforms, and precise transcriptional and translational regulation options. Investigation of the scope of employing codon-optimized Cas9 and modified Cas9, like high-fidelity Cas9 (SpCas9-HF1), enhanced-specificity Cas9 (eSpCas9), and hyper-accurate Cas9 (HypaCas9) in RTC genome editing may help produce excellent results in terms of specificity and efficiency. Genome editing-based RTC improvement programs can also benefit from recent innovations in CRISPR/Cas9-dependent base editing and prime editing techniques. Validating the conserved mechanisms of the cell’s choice of the DSB repair pathway or elucidating the molecular mechanisms that stimulate HDR in RTC species and channeling these mechanisms toward the HDR pathway will enhance the efficiency of CRISPR/Cas9-mediated gene insertion for introgression of the novel traits into the target RTC. Methods such as the suppressing of the NHEJ pathway by RNA silencing or transcription inhibition mechanisms, stimulating HDR-promoting factors like RAD51 recombinase, employing modified Cas9 to produce nicks instead of DSBs, and temporally specifying transformation during cell division when the HDR mechanism is active can promote the HDR repair pathway (Pinder et al., 2015; Nambiar et al., 2019). Since the regeneration and transformation of many RTCs is still a bottleneck, the identification of appropriate media composition for regeneration and standardization of suitable transformation protocols for major and underutilized RTC species are crucial for the success of gene editing applications. Successful CRISPR/Cas RNP delivery and regeneration of edited plants have been observed in potato, while an efficient protocol is yet to be optimized for other RTC species. This aspect has to be treated with sufficient importance, as it is the key factor facilitating non-transgenic editing.

Robust multiplexing options provided by CRISPR/Cas9 are particularly beneficial for RTC species because of heterozygosity, polyploidy, and the multi-gene family regulation of certain economically important traits. This is particularly important in stress resistance, as evidenced in the case of virus resistance conferred by the multiplex editing of the target gene. The dosage effect of alleles governing multiallelic traits can be analyzed through multiplex editing. This is observed in experiments with *ALS* and *PPO* gene editing, in which single and multiple allele editing revealed their single allelic regulation on herbicide resistance and tuber browning, respectively. Thus, it is obvious that multiplex editing can provide precise information regarding the targets to be edited. The feasibility of spatiotemporally specified editing using CRISPR/Cas cassettes organized with germline-specific promoters or meiosis-specific promoters should be evaluated since this is valuable for ensuring the inheritance of induced mutations in T1 generation itself (Eid and Mahfouz, 2016; Mao et al., 2016). Optimization of delivery of gRNA coupled with germ cell homing mRNA strategy for RTC species should be considered an important objective for future research. This approach will ensure the inheritance of mutations.

## 6 Conclusion

Various studies reported so far substantiate the utility of CRISPR/Cas9 in achieving RTC improvement concerning stress resilience, value addition, biofortification, and quality refinement. Genome sequencing, transcriptome profiling, and functional analysis through reverse genetics approaches have identified genetic components that could be manipulated to achieve target traits in certain RTC species. CRISPR/Cas can also be utilized to enhance gene functional analysis in RTCs. The robust editing platform and non-transgenic strategy offered by CRISPR/Cas enable the efficient application of these databases to engineer crop genomes in alignment with both previous and novel findings to develop RTC landraces with desirable attributes.

Although CRISPR/Cas appears to be a highly promising technique for crop improvement, factors like off-target effects, collateral effects, and regulatory concerns are major barriers to its widespread application. So, further research in genome editing techniques is necessary to overcome these hurdles. Additionally, studies to optimize allied strategies that include transformation and regeneration, *in vitro* propagation, delivery systems, acclimatization, and proper maintenance of genome-edited lines are crucial. This ensures the availability of planting material of improved RTC varieties to farmers at low cost and in sufficient quantity, thereby bringing the benefits of research and technology development to the field level.
